# *In silico* design of the first DNA-independent mechanism-based inhibitor of mammalian DNA methyltransferase Dnmt1

**DOI:** 10.1371/journal.pone.0174410

**Published:** 2017-04-11

**Authors:** Vedran Miletić, Ivica Odorčić, Patrik Nikolić, Željko M. Svedružić

**Affiliations:** 1 Molecular Biomechanics Group, Heidelberg Institute for Theoretical Studies (HITS), University of Heidelberg, Heidelberg, Germany; 2 Laboratory for Biomolecular Structure and Function, Department of BioMedical Technology, Center for Advanced Computing and Modelling (CNRM), University of Rijeka, Rijeka, Croatia; University of Parma, ITALY

## Abstract

**Background:**

We use our earlier experimental studies of the catalytic mechanism of DNA methyltransferases to prepare *in silico* a family of novel mechanism-based inhibitors of human Dnmt1. Highly specific inhibitors of DNA methylation can be used for analysis of human epigenome and for the creation of iPS cells.

**Results:**

We describe a set of adenosyl-1-methyl-pyrimidin-2-one derivatives as novel mechanism-based inhibitors of mammalian DNA methyltransferase Dnmt1. The inhibitors have been designed to bind simultaneously in the active site and the cofactor site and thus act as transition-state analogues. Molecular dynamics studies showed that the lead compound can form between 6 to 9 binding interactions with Dnmt1. QM/MM analysis showed that the upon binding to Dnmt1 the inhibitor can form a covalent adduct with active site Cys1226 and thus act as a mechanism-based suicide-inhibitor. The inhibitor can target DNA-bond and DNA-free form of Dnmt1, however the suicide-inhibition step is more likely to happen when DNA is bound to Dnmt1. The validity of presented analysis is described in detail using 69 modifications in the lead compound structure. In total 18 of the presented 69 modifications can be used to prepare a family of highly specific inhibitors that can differentiate even between closely related enzymes such as Dnmt1 and Dnmt3a DNA methyltransferases.

**Conclusions:**

Presented results can be used for preparation of some highly specific and potent inhibitors of mammalian DNA methylation with specific pharmacological properties.

## Introduction

DNA methylation is a fundamental mechanism in functional organization of the human genome. DNA methylation is one of the first steps in epigenetic regulation and the most enduring epigenetic landmark [1]. Inhibitors of DNA methylation can be used in studies of human epigenome [2], or in biomedical technology for creation of induced pluripotent stem cells (iPSC) and cellular reprogramming [3–5]. Specific inhibitors of DNA methylation offer several advantages over knockdown studies. First, the inhibitors do not disrupt multimolecular complexes that form around DNA methyltransferases in mammalian cells [6]. Second, inhibitors can be used in very precise dose-dependent and time-dependent protocols. Inhibitors of DNA methylation can be also used in clinics for treatment of oncogenic transformation, viral infections, immunological disorders, or neurological and psychiatric impairments [7–11].

Dnmt1 is the principal DNA methyltransferase in mammalian cells [6, 12]. More than 20 different inhibitors of mammalian Dnmt1 have been described in the last 30 years [13]. Unfortunately, none of those studies gave consistent results and sustainable progress [11, 13]. The observed inconsistencies and the lack of progress could be in a large part attributed to assay design and regulation of Dnmt1 activity in cells. Dnmt1 in cells can interact with about 40 different proteins and with some RNA molecules [6, 14, 15]. Thus, DNA methylation in cells can be affected by any change in DNA metabolism, DNA repair, chromatin organization, and cell cycle control [6, 14]. Cell-based studies of inhibition of DNA methylation cannot differentiate between compounds that target Dnmt1 directly from the compounds that can stall DNA methylation by causing DNA damage or other changes in DNA structure and metabolism. Thus, the screenings for inhibitors have to start with purified Dnmt1, and then well-characterized compounds can be used to study inhibition of DNA methylation in cells. The screenings with purified Dnmt1 have to be designed to differentiate between compounds that bind to Dnmt1 from the compounds that interfere with DNA methylation by binding to the DNA substrate. None of the published studies of inhibition of DNA methylation have included all of the required precautions, and still none of the published studies found compounds with IC50 values significantly below 1 μM [10, 13].

Mechanism-based inhibitors can give the highest specificity and thus lowest toxicity [16]. Different cytosine derivatives are the only true mechanism-based inhibitors that have been developed in the last 40 years [13]. The cytosine derivatives were introduced based on mechanistic similarities between DNA methyltransferase and thymidylate synthase [17]. The cytosine derivatives have been very useful in mechanistic studies of DNA methyltransferases [18–20], however their full applicability as inhibitors of DNA methylation is very limited due to high toxicity in cells [13]. Very little improvement has been introduced following the initial studies, mostly due to the unusually challenging mechanism of the target base attack in the process of DNA methylation [12, 18–22]. Consistent with earlier activity studies recent crystal structures showed that the target base attack depends on multiple flexible loops in the protein structure [18–21, 23]. Regulation of protein function by flexible loops in protein structure can be described very effectively by combining experimental and computational approaches [24, 25].

In this study we present a novel strategy to design a whole family of mechanism-based suicide-inhibitors. The inhibitors are designed to act as a transition-state analogue by binding simultaneously to the AdoMet site and the active site on Dnmt1 [16]. The combination of two binding sites and the mechanism-based suicide-inhibition was chosen to provide highly potent and highly specific inhibition of DNA methyltransferases [16]. The presented strategy is a combination of insights from extensive enzyme activity studies, recent crystal structures, and some of the most recent computational approaches [18–21, 24].

## Transition-state analogue as a lead compound for a mechanism-based suicide-inhibitor

**Fig 1:**
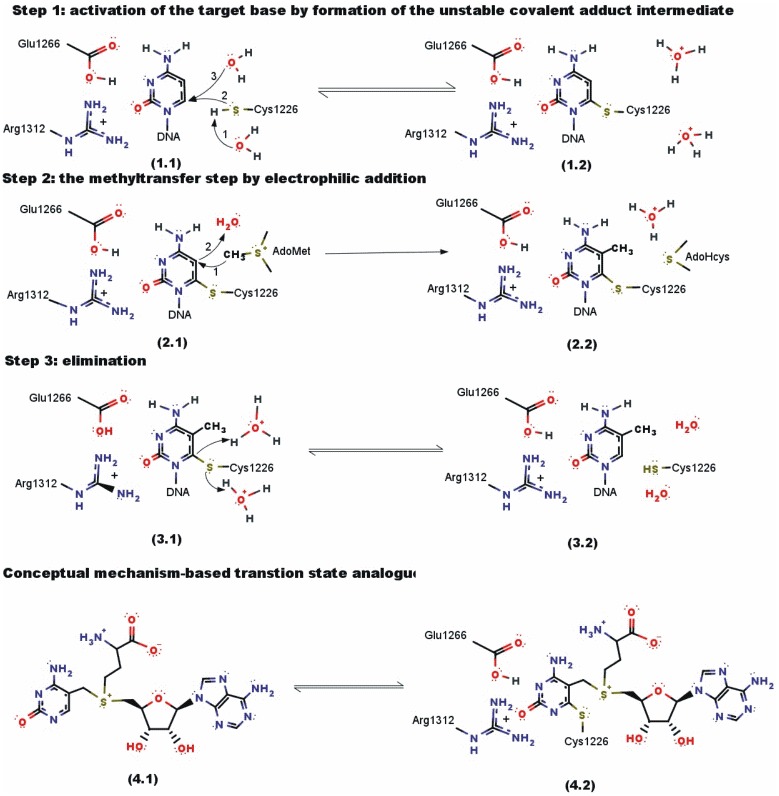
Catalytic mechanism of DNA methyltransferase Dnmt1. The catalytic mechanism of bacterial and mammalian cytosine-C5 DNA methyltransferase has been described in details in the last 40 years in different crystal structures, enzyme kinetics studies, enzyme substrate binding studies, and QM/MM studies [17–21, 29, 34]. The catalysis consists of three main steps. The catalysis is initiated when the aromatic target base ring is posited in the active site cavity (step 1.1). In the case of human and mouse enzymes, the active site is composed of conserved Arg 1310, Arg 1312, Glu1266, and Cys 1126 residues (step 1.1). The enzyme can form unstable covalent adduct intermediate when conjugated aromatic bonds in the target base ring are positioned in an asymmetric active site between polar amino acids (steps 1.1 to 1.2). Kinetic and QM/MM studies showed that formation of the covalent adduct intermediate is a reversible step unless the reaction is driven forward by the rate-limiting irreversible methyltransfer step [19, 20, 29]. Following the methyltransfer step (steps 2.1 to 2.2), there is a reversible target base eliminations step (steps 3.1 to 3.2). We have decided to target this reversible step with a mechanism-based suicide-inhibitor. The structure 4.1 is supposed to mimic the transition from structures 2.1 to 2.2. Thus, when structure 4.1 is positioned in the active site it should trigger the formation of the covalent adduct shown as the structure 4.2. This is in essence, the same process as the transition from structure 3.2 to structure 3.1. The figures were drawn in ChemAxon Marvin 15.0.1 [39]

**Fig 2:**
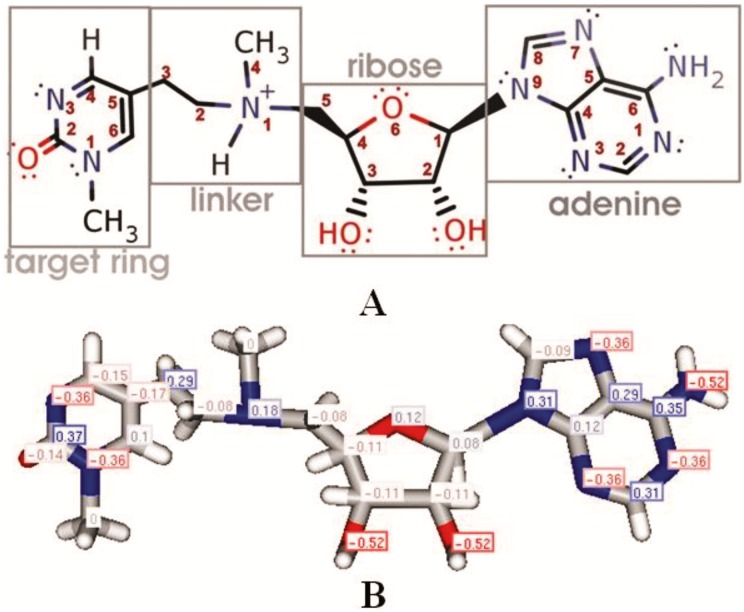
(A-B) Lead structure for the mechanism-based suicide-inhibitor of Dnmt1. (**A**) The figure shows our first prototype for a mechanism-based inhibitor of Dnmt1. The structure is derived from the structure 4.1 in Fig 1 as a reasonable challenge for organic synthesis that has a reasonable agreement with the Lipinski's rules [26, 27]. The structure is designed to act as a transition state analogue and consists of four functional parts: adenine, ribose, tertiary amine linker and 1-methyl-pyrimidin-2-one as the target base ring. The adenine and ribose parts are selected to assure that the lead compound can bind to AdoHcys/AdoMet sites. Tetrahedral tertiary amine linker is chosen as a flexible structure that can mimic the transition state structure (Fig 1). 1-methyl-pyrmidine-2-one was chosen as the target base ring instead of cytosine, to avoid problems with deamination at carbon 4 and to decrease polar surface on the target base ring [19, 22]. (**B**) The present structure is in agreement with the Lipinski's rules except for its low LogD value. The relative molecular mass is 417.45 Da. There are 6 rotatable bonds, and a total of 58 bonds between 55 atoms. At pH 7.40 the polar surface area is 156.42 Å^2^ while Van der Walls surface area is 562.17. There are five H-bond donor sites and 13 acceptor sites. At pH 7.40, 94% of the ligand is protonated at the linker nitrogen and the compound is positively charged. The LogD value in protonated form is -2.63 and -1.88 in unprotonated form. To improve ADME properties the future modifications will be designed to increase the LogD value. This can be achieved by removing the positively charged nitrogen at the linker, and by removing heteroatoms at the ribose and adenine ring that give the highest negative contribution to the LogD value. The numbers on the structure show contribution to the total LogD for each atom. All parameters have been calculated using ChemAxon Marvin suite version 15.3.16 [39]

The structures 4.1 and 4.2 shown in Fig 1 illustrate the concept of mechanism-based suicide-inhibition. Fig 2 shows a realistic functional derivative of the conceptual structure 4.1. in Fig 1 [26, 27]. In essence, our mechanism-based inhibitor is designed to mimic the last elimination step in the catalytic cycle of DNA methyltransferases (Fig 1). This concept was first presented in our earlier paper on catalytic mechanism of Dnmt1 [12]. Subsequently, a very similar concept was used to prepare novel inhibitor of histone methyltransferase DOT1L [28].

**Fig 3:**
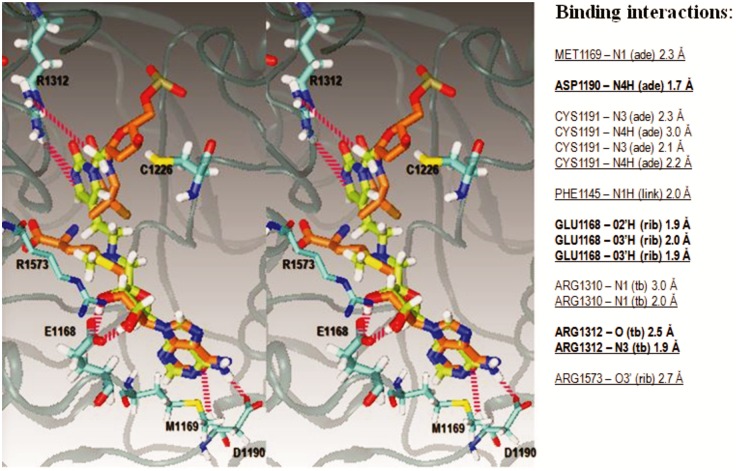
MM/MD simulations of binding interactions between our prototype inhibitor and Dnmt1 (PDB: 4DA4 [18]). (**A**) The cross-eyed stereo image shows that our lead compound can bind to Dnmt1 in the same position as AdoHcy and the target cytosine in crystal structures of Dnmt1 (PDB: 4DA4 [18]). The image shows the lead compound (lime green), the interacting amino acids (cyan) and the crystal structure of AdoHcys (pink) and the target cytosine (pink) [41]. Hydrogen atoms are shown in white, oxygen in red, nitrogen in blue, and sulfur in yellow. The figure was prepared by superimposing one of the MM/MD simulation frames of Dnmt1-inhibitor complex to the crystal structure of Dnmt1 in complex with AdoHcys and the target cytosine (PDB: 4DA4 [18]). The table on the left gives a full list of all possible binding interactions that can be observed in MM/MD simulations. The most stable interactions are shown in bold. For clarity, the figure does not show binding interactions with Cys1191, Phe1145, Trp 1170, Arg 1310 or the surrounding solvent molecules. All of the interacting amino acids are conserved between mouse and human Dnmt1 [21].

MD simulations confirmed that our lead-compound can bind simultaneously at the cofactor binding site and at the active site (Fig 3 and S1 Movie). In this position, the inhibitor can act as a transition state analogue. Precisely, adenine and ribose parts of the inhibitor overlap with corresponding AdoHcys parts in crystal structures (Fig 3). The target base ring can overlap with corresponding 5-methyl-cytosine parts in crystal structures (Fig 3). The ethyl branch on the linker overlaps with the 5-methyl group on the target cytosine in crystal structure (Fig 3 and [18]). Thus, the linker is in position of expected reaction coordinate where it can mimic the rate-limiting methyltransfer step, i.e. transition state intermediate (Fig 1 and [29]). The tertiary nitrogen is positioned in the same place as a sulfur atom in AdoHcys, while its methyl-tail is positioned in the same place as the homocysteine tail of AdoHcys (Fig 3).

**Fig 4:**
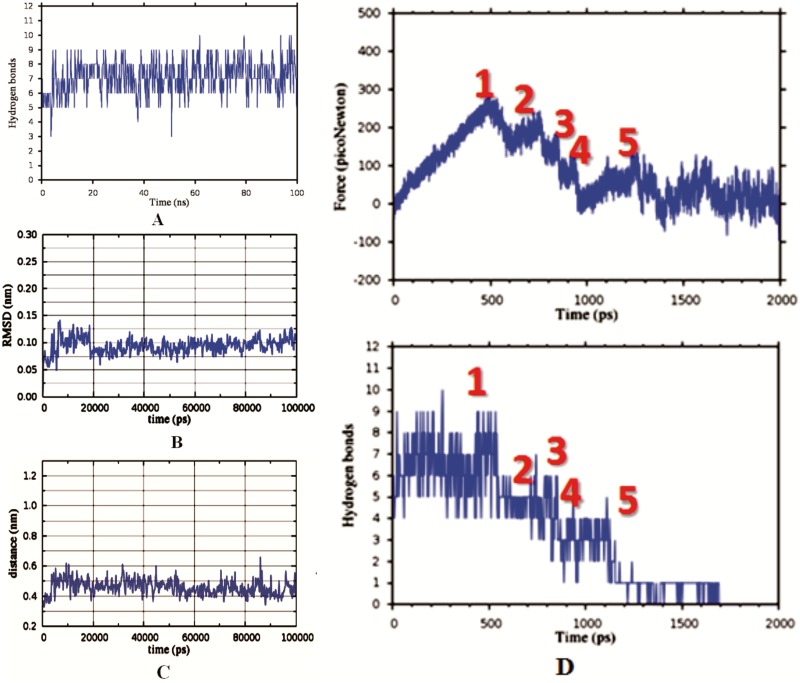
(A-D) MM/MD simulations of binding interactions between our prototype inhibitor and Dnmt1 (PDB: 4DA4 [18]). Dynamic binding interactions within Dnmt1-linhibtor complex depicted in Fig 3 can be analyzed by following changes in “hydrogen-bond” plots (A), “average-ligand-RMSD” plots (B), “Cys1226-carbon-6-distance” plots (C), and “inhibitor pulling force” plots (D). **(A)** In average 7±2 hydrogen bonds can be observed in Dnmt1-inhibitor complex in 100 nsec simulations (S1 Movie). **(B)** Distinct steps and peaks in RMSD plots represent different conformations of the ligand within the complex, while uniform RMSD plots represent limited ligand mobility and one dominant conformation in a stable complex ([37, 38] and S1 Movie). **(C)** Distinct steps and peaks in “Cys1226-carbon-6-distance” plot represent different swiveling motions by the target base in the active site cavity (S1 and S3 Movies). **(D)** Steered molecular dynamics protocol was used to calculate “inhibitor pulling force” plots and the corresponding changes in binding interactions (S2 Movie). Changes in the pulling force that correspond to different dissociation steps are labeled with numbers. The numbers indicate: (1) force required to initiate movement of the inhibitor in its binding site and the resulting opening of active site loop; (2) force required for full displacement of the target base; (3–4) force required for full dissociation of the ribose ring from Glu 1168 and Arg 1573 respectively; (5) force required for full dissociation of the adenine ring.

MD simulations showed that the inhibitor makes in average 7 ± 2 dynamic hydrogen bonds with the enzyme (Fig 4, GROMACS default criteria for hydrogen bond, 3.5 Å and 30-degree angle [30, 31]). We could count up to 15 binding interactions using different protocols and different simulation frames (Fig 3). All of the interacting amino acids are conserved between mouse and human Dnmt1 [21]. The four functional parts on the inhibitor give a different contribution to binding interactions (Fig 4D, and S2 Movie). The target base ring is supported in the active site by two ion-dipole interactions between its carbonyl oxygen and Arg 1310 or Arg 1312 (Fig 3). The linker section of the inhibitor can form 1.95 Å long hydrogen bond between its tertiary nitrogen and peptide backbone of Phe1145. Rest of the linker makes no binding interactions with the protein surface but remains enclosed in 3.7 Å long groove by the active site loop (amino acids 1220 to 1236). In some rare instances wobbling of the linker can drive the target base out of the active site groove. The corresponding simulation frames can be detected as extremes on “Cys1226-carbon-6-distance” plots (Fig 4D and S2 Movie).

Ribose makes several binding interactions that are so strong that they keep the inhibitor bound to the enzyme even when the target base ring and the linker are fully dissociated (S2 Movie). First, the H atoms on 2’ and 3’ OH groups make 1.85 and 1.91 Å long hydrogen bond with the negatively charged Glu1168 [18, 21, 32]. Both -OH groups and Glu1168 can make dynamic hydrogen bonds with the bulk solvent molecules, however such interactions do not affect the stability of the complex [18, 21, 32]. Interestingly, we found that the oxygen atoms on the two OH groups can form 2.7 and 3.0 Å long ion-dipole interactions with Arg1573. Those interactions are not visible in crystal structures. Precisely, the interactions with Arg1573 can be observed only in MD simulations with rotamers that swivel towards the ribose ring and form π-π stacking interaction with Trp1170 (S2 Movie).

**Fig 5:**
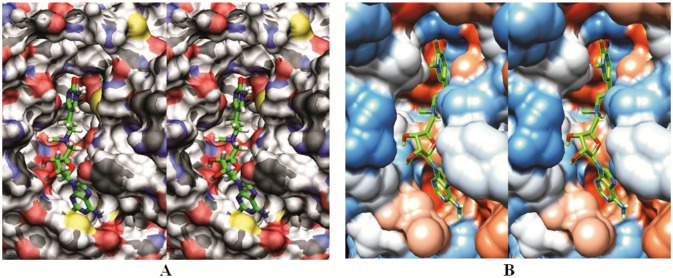
(A-B) Binding of the prototype inhibitor to its binding site groove on Dnmt1. The two cross-eyed stereo images show the lead compound bound to mouse Dnmt1 as in Fig 3, except that the binding site is shown in the surface mode to highlight its shape, depth, and hydrophobic patches. (**A**) image shows surface hydrogens in white, carbons in black, nitrogens in blue, oxygens in red, and sulfur in yellow [41]. The colors can help in tracing the amino acids shown in Fig 3 on the groove surface. (**B**) the image shows hydrophobic surface in brown, and hydrophilic surface in blue [37, 38]. In both images, the prototype inhibitor is shown in stick model and colored green. Reader is looking at the binding site cleft from the position of DNA substrate, therefore methyl group on nitrogen 1 of the target base ring is positioned almost perpendicular to the plane of paper. The adenine ring is positioned in a tight hydrophobic surface pocket enclosed by Met1169, Asn1190 and Cys1191. The ribose ring is anchored on a wide ridge with its two OH group leaning atop of Glu1168. The linker is positioned in the widest section of binding site cleft just above AdoHcys/AdoMet binding cavity. The target base ring is positioned in the active site cavity and its carbon 6 is in Van der Waals contact with Cys1226.

The adenine ring sits in a tight hydrophobic pocket behind Met 1169 (Figs 3–5, and ref. [18]). The adenine is stabilized by three highly dynamic hydrogen bonds between N3 and N4 atoms and Asp1190 or Cys1191 in protein backbone (Fig 3, and S1 and S2 Movies).

## Formation of a covalent adduct between the target base and the active site Cys 1226

**Fig 6:**
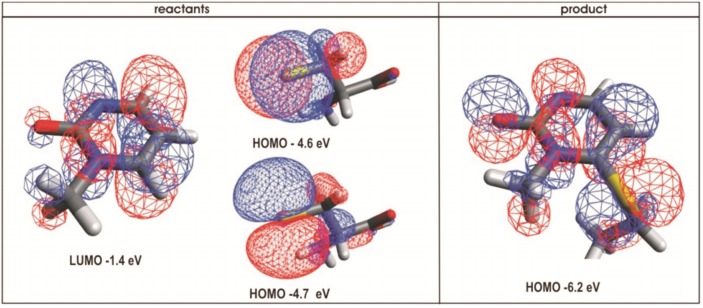
Frontier molecular orbitals on the target base and on the active site cysteine 1226. The LUMO orbitals on carbon 6 of the target base ring are perpendicular to the plane of the ring. The two HOMO orbitals on nucleophilic sulfur anion are perpendicular to each other and have almost identical energy. The reaction can start when one of the two HOMO orbitals on the sulfur atom comes in the plane of the target base ring (S3 Movie). The orbitals and the corresponding energies were with program GAMESS [42] using DFT B3LYP protocol and visualized in VMD [41]. The bond forming motions can be driven by the by the rotation of the target base ring around its hydrogen bonds axis with Arg 1312 (S3 Movie).

The key step in mechanism-based suicide-inhibition is the formation of a covalent adduct between the active site Cys 1226 and the target base on the inhibitor (Fig 1). The suicide-inhibition can make our inhibitor highly potent and highly specific for Dnmt1 [16]. We used QM/MM studies to show that the inhibitor can make a covalent adduct with the active site Cys 1226 after binding to Dnmt1 (Figs 3–6).

Analysis of frontier orbitals showed that the active site Cys 1226 can form a covalent adduct with carbon 6 on the target base when the reactive atoms come in the same plane (Fig 6). MD and QM/MM analysis show that such event depends on two sets of conformational changes (S2 and S3 Movies). First, the constant swiveling of the target base around its hydrogen bond with Arg 1312 has to drive the carbon-6 on the target base ring and Cys 1226 in the same plane (Fig 6, S3 Movie). In the second set of the conformational changes the active site loop has to be driven in its closed position, so that Cys1226 is forced into a tight contact with the carbon 6 on the target base (S4 Movie). MD simulations showed that distances between active site Cys 1226 and carbon 6 on the target base can vary between 3.5 and 6 Å, with an average distance of 4.7 Å (Fig 4). Our QM/MM studies showed that distances of 4 Å can readily support the formation of a covalent adduct between the target base and Cys 1226 (S3 Movie). Similar reactive distances have been reported in the earlier studies [29].

QM/MM studies also showed that similar to the previous studies [29], the lowest activation energy for the formation of a covalent adduct can be observed when two reactive hydroxy groups are present in the active site cavity (S3 Movie, and manuscript in preparation). The swiveling on the target base that is driving the reaction is not as noticeable in the S1 to S3 Movies. This can be expected. The molecular events described in the S3 Movie require recording with much faster frame rate than the events described in the S1 and S2 Movies.

## Binding of the prototype inhibitor can be affected by conformational changes in the active site of Dnmt1

**Fig 7:**
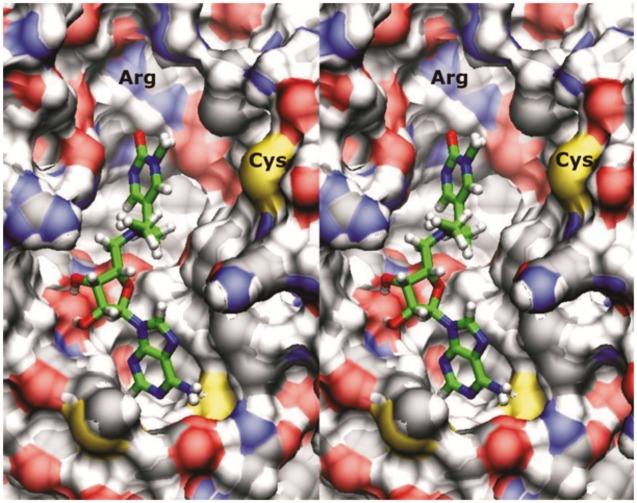
The cross-eyed stereo image shows the inhibitor bound to the crystal structure of Dnmt1 with the active site loop open (PDB code 3AV6, [32]). [41]. The inhibitor is docked in its binding site just as in Figs 3 and 4, except that the active site is open (amino acids 1220 to 1236). All binding interactions are still present, except that the calculated “Cys1226-carbon-6-distance” is 9.2 Å. Thus the inhibitor can bind to Dnmt1 when its active site is in open and closed position, however mechanism-based suicide-inhibition can happen only when the active site loop is closed.

We further analyzed how conformational changes in the active site of Dnmt1 can affect the inhibition (Fig 7, and S4 Movie). Specifically, the active site loop swings between open and closed position when Dnmt1 is in DNA-free from [18–21, 32]. The loop is closed when DNA and the cofactor form a catalytic complex [18]. DNA-bound and DNA-free forms of Dnmt1 represent different stages in the physiology of DNA methylation [6]. Thus, the loop related changes in inhibitor activity can define pharmacological properties of our inhibitors.

We started steered molecular dynamics analysis of Dnmt1-inhibitor complex with the active site loop is in closed position as depicted in Fig 5 (S4 Movie, PDB code: 4DA4 [18]). Surprisingly the simulations showed that gradual opening of the active site loop does not lead to immediate dissociation of the target base ring and the inhibitor. The opening of the active site loop leads to increase in mobility in the linker and the target base which allows more favorable interactions between the target base ring and Arg1310 and Arg1312. The opening of the active site loop primarily leads to increase in “Cys1226-carbon-6-distance”. Active site Cys 1226 is located at the flexible tip on the loop (S4 Movie, PDB code: 4DA4 [18]). Taken together these results indicate that the inhibitor can bind to Dnmt1 when the active site is open and closed. However, the suicide-inhibition is fastest when the active site loop is closed, for example when Dnmt1 is bound to hemimethylated substrate in the process of maintenance methylation (PDB code: 4DA4 [18]). Much slower suicide inhibition can be expected when Dnmt1 is bound to unmethylated DNA (PDB code: 3PT6 [21]) or when Dnmt1 is in its DNA-free form (PDB code: 3AV6 [32]). In those cases, the rate of suicide inhibition will depend on slow thermal swinging between open and closed position [20].

## Modifications in the lead-compound structure and related changes in binding interactions

We have analyzed 69 modifications in the lead compound structure with a desire to create a family of mechanism-based inhibitors that could have different pharmacological properties (S1–S5 Tables). The modifications have been designed to change ligand flexibility or to replace some of the interacting groups with other groups that can provide similar interactions. The changes in binding interactions caused by different modifications can give additional insights into the binding mechanism and also provide a good test of the reliability of the presented analysis of Dnmt1-inhibitor complex. In total, we found that 18 of presented 69 modifications can bind to Dnmt1 like a transition state analogue (Fig 3), give favorable LogD values, and show “Cys1226-carbon-6-distance” that can support mechanism-based suicide-inhibition (S1–S5 Tables, compound numbers shown in red). In choosing some of the modifications we have followed experimental insights from studies of different AdoMet analogues and Dnmt1 [19, 20, 33].

### Modifications in the adenosine ring

We have analyzed eight modifications in the adenine ring (S1 Table). Three of these modifications give favorable LogD values and “Cys1226-carbon-6-distance” plots. The prepared modifications can be separated in two sets. The first set are modifications at the carbon 6 amino group (S1 Table), and the second set are modifications within the ring structure (S1 Table).

The amino group on carbon 6 is positioned on a hydrophobic ridge at the edge of the adenosine binding pocket where it makes a maximum of three hydrogen bonds with Asp 1190, Cys1191 and bulk solvent molecules (Figs 3–5). We have replaced the amino group with hydrogen atom, nonpolar methyl group, or carbonyl group (S1 Table). The binding was not observed only when a hydrogen atom was placed on carbon 6, what is suggesting that a group of a certain size is needed to guide the adenosine ring in its binding ridge (Figs 3 and 5). Both methyl group and carbonyl groups support binding, which indicates that the hydrogen bonds with an amino group on carbon 6 are not crucial for binding. Interestingly, MM/MD simulations showed that the carbonyl group can reposition modified ring in the ligand binding cavity so that optimal interactions with Lys1244 can be achieved. Such repositioning can increase the ligand RMSD values however the “Cys1226-carbon-6” distance plots show that both modifications can support complex that could initiate the suicide-inhibition step (S1 Table).

Next, we introduced modifications within the ring structure (S1 Table). We made the ring more hydrophobic by replacing polar nitrogen atoms with carbon atoms or by replacing the aromatic ring with a saturated ring (S1 Table). We find that increase in hydrophobic surface can bury the modified ring in its binding pocket and decrease the ligand mobility in its binding site groove (Figs 4 and 5, and S1 Table). This results in a decrease in RMSD values, and in a very favorable decrease in “Cys1226-carbon-6” distance plots (S1 Table). The only exception is a fully saturated ring, which is too big to slip into the binding site pocket that is suited for a flat aromatic ring. The ligand with the saturated ring can dock in the binding site groove, but corresponding RMSD and “Cys1226-carbon-6” distance plots show that the ligand with the saturated ring is only loosely docked in the binding site groove and cannot support the suicide inhibition step (Fig 6).

### Modifications in ribose part of the inhibitor

We have analyzed 20 modifications in the ribose ring and found that only two of them give favorable LogD values and “Cys1226-carbon-6-distance” plots. Modifications have been prepared in all five positions on the ribose ring (S2 Table).

Modifications in place of the two -OH group are most attractive since the -OH groups give the highest contribution to binding interactions and to the LogD value (-0.52 per one OH group, Fig 2). We find that our lead compound cannot bind to Dnmt1 in desired orientation when the two -OH groups are replaced with H atoms (S2 Table). The ligand can bind in the desired position when the OH group on carbon 3 is kept and OH group on carbon 2’ is replaced with H atoms. However, there is no binding when the OH group on carbon 2’ is kept and OH group on carbon 3’ is replaced with H atoms. The MM/MD simulations show that the OH group on position 3’ has more influence on binding than the OH group on position 2’, since it has more favorable interaction geometry with Glu 1168 and tighter interactions with the walls of the binding site groove. Placing a double bond between 2’ and 3’ carbon atoms will not support binding of the inhibitor. With a double bond, the two OH groups are lifted in the plane of the ribose ring where they cannot make binding interactions with Glu 1168 or Arg 1573.

Next, we replaced the two -OH groups with -F or -Cl atoms (S2 Table, compounds 16 to 21). We find that fluorine atoms in α-position support binding and favorable “Cys1226-carbon-6” distances. Binding is also observed when the two F atoms are placed in the plane of the ring by a double bond. Fluorine atoms placed in β-position support binding, but do not support conformations that can give favorable “Cys1226-carbon-6” distances. Chlorine atoms support binding in only two positions and none of those positions can support conformations that could give favorable “Cys1226-carbon-6” distances. In all complexes with -F or -Cl modifications negatively charged Glu 1168 rotates away from the ribose ring, while positively charged Arg 1573 is positioned about 2.9 Å above the ribose ring where it makes unstable π-π stacking interactions with Trp 1170.

The ligand binding is also observed when both -OH groups are removed and the carbon 3’ is replaced with an oxygen atom (S2 Table). MM/MD simulation showed that such protein-ligand complex is very unstable since its binding is supported by a water molecule in α–position that is bridging between the oxygen at 3’ and the Glu 1168. Surprisingly, we could not find a replacement for the acetal oxygen at the 5’ position of the ring (S2 Table). The acetal oxygen is in direct Van der Waals contact with Phe1145. Thus any group at that position that is bigger than an oxygen atom can push the ribose ring out of its binding groove.

### Modifications in the linker structure

We have analyzed 19 modifications in the linker, 5 of those give favorable LogD values and “Cys1226-carbon-6-distance” plots (S3 Table). The linker makes only one binding interaction with binding site surface, its tertiary nitrogen makes 2.17 Å long bond with peptide backbone on Phe1145 (Fig 3). Thus, modifications in the linker have been primarily designed to facilitate synthesis of inhibitor or to decrease inhibitor’s flexibility and LogD value (Fig 2). We analyzed linkers with oxygen, sulfur, and carbon in the place of tertiary nitrogen (S3 Table, compounds 39, 43, 44, 46, 47). Simulations showed that linkers with oxygen and sulfur atoms gave favorable LogD values and “Cys1226-carbon-6-distance” plots (compounds 43 and 44). A linker with a—CH_2_- group also supports binding of the inhibitor. However, the binding conformations do not support optimal orientation of the target base in the active site cavity since hydrophobic—CH_2_- cannot lean on to hydrophilic surface formed by peptide backbone of Phe1145 (Fig 5B). Encouraged by favorable features observed with the linker with an oxygen atom (compound 43), we tested two more linkers with oxygen atom modifications (S3 Table, compounds 46 and 47). Compounds 46 and 47 gave favorable LogD values and “Cys1226-carbon-6-distance” plots (S3 Table).

The methyl tail on linker can stabilize Dnmt1-inhibitor complex to some degree by anchoring the linker in the hydrophilic AdoMet cavity (Fig 5B). However, these effects are very limited since the tail can also push the linker out of the binding site by repeated collisions with the binding site surface. The repulsive collisions with protein surface are best visible when the methyl tail is replaced with longer tails such as hydroxymethyl or ethyl tail (S3 Table).

Next, we placed double bonds or methyl groups in different positions on the linker in an attempt to analyze how changes in linker flexibility can affect binding interaction (S3 Table). We find that two *entgegen* double bonds in position 1–2 and 3–4 on the linker can give exceptionally steady RMSD plots and much more favorable “Cys1226-carbon-6-distance” than the lead compound (S3 Table, compound 42). MM/MD simulations show that compound 42 appears to be perfectly molded for the binding site groove. Compound 37 is another example of a compound with a double bond in the linker that gives favorable LogD values and “Cys1226-carbon-6-distance” plots. Compound 48 is an example of a modification that gave favorable “Cys1226-carbon-6-distance” by selectively placed methyl groups on the linker. The methyl groups on the linker can wedge the inhibitor in its binding site groove.

### Modifications in the target base ring

We have analyzed 8 modifications in the target base ring, three of those give favorable “Cys1226-carbon-6-distance” plots. Some electron-withdrawing modifications on the target base ring could increase the rate of nucleophilic attack by the active site Cys 1226 and enhance stability of the resulting covalent adduct (Fig 6 and S3 Movie). Such modifications can be quantitatively compared by following the energy of LUMO orbitals on the target base ring (S4 Table). For example, -CF_3_ and -CFH_2_ groups on the nitrogen 1 can decrease the energy of LUMO orbitals on the target base ring. MM/MD studies showed that inhibitors with -CFH_2_ group on the nitrogen 1 can bind and give more favorable “Cys1226-carbon-6-distance” plots than the lead compound. The binding is stabilized in part by interaction between the fluorine atom and Arg1573. MM/MD simulations showed that target base ring with -CF_3_ group on the nitrogen 1 cannot give favorable “Cys1226-carbon-6-distance” plots, since -CF_3_ is too big to fully fit in the active site cavity with the active site loop closed (Fig 7). Interestingly, we also found that electron-withdrawing modifications at the nitrogen 1 can facilitate the synthesis of the inhibitor by making the target base more resistant to side reactions during oxidation steps.

The rate of nucleophilic attack by the active site Cys1226 can also be affected by modifications that can influence the wobbling of the target base in the active site cavity (S3 Movie, manuscript in preparation). 1,4-dimethyl-pirimidin-2-one is too bulky to fit in the active site cavity. On the other hand, pirimidin-2-one is too small to fill the active site cavity what results in excessive mobility that can interfere with productive collisions with Cys 1226. 1-Methyl-pirimidin-2-one is the best choice for the target base ring and mechanism-based suicide-inhibition (S3 Movie).

1-Methyl-cytosine makes more binding interactions with the active site residues than any other modification. Precisely, the amino group on cytosine carbon 4 can form hydrogen bonds with Glu1266 in the active site [18]. However, for several reasons, 1-methyl-pirimidin-2-one is a better choice for the target base than 1-methyl-cytosine. First, the amino group on carbon 4 is known to hydrolyze and turn into carbonyl group which has a repulsive interaction with Glu1266 (compound 57 and [20]). Second, 1-methyl-pirimidin-2-one makes a stable covalent adduct that can be even captured in crystal structures [29, 34], while cytosine makes unstable and readily reversible covalent adduct [19, 20, 29]. 1-Methyl-pirimidin-2-one lacks the electron rich amino group, so it is a better electrophile than cytosine (its LUMO is -1.4 eV, vs. LUMO for cytosine is -0.9 eV). Finally, inhibitors with 1-methyl-pirimidin-2-one are easier for synthesis than 1-methyl-cytosine due to lesser reactivity in side reactions [26, 27].

### Modifications that can mold the inhibitor to its binding site groove

We also attempted to wedge the inhibitor in its binding site groove by preparing parallel modifications at the ribose ring and the linker at the sites that have been thus far the most difficult to modify (S5 Table). First, methyl groups have been placed on ribose and on linker in attempt to wedge the inhibitor in its binding site groove (Fig 5). Second, double bonds and longer ethyl tail were placed on the linker to restrict its conformational space in the binding site groove. We found that a methyl group in β–position on carbons 2’ and 3’ on ribose ring can wedge the inhibitor in its binding site groove in desired conformation (S5 Table, compounds 60–61, 65–66). Such approach makes the Dnmt1-ligand complex less dependent on anchoring interactions between—OH groups on ribose ring and Glu1168 on protein surface. Ribose molecules with methyl groups in β–position on carbon 2’ and carbon 3’ are commercially available from several suppliers.

## Binding specificity: Human Dnmt1 vs. human Dnmt3a DNA methyltransferase

Mechanism-based inhibitors can provide the highest specificity for the target enzyme [16]. However, mechanism-based inhibition might not be enough to ensure desired specificity in our case since adenosyl part on our inhibitor could bind with significant affinity to a large number of cellular enzymes that bind similar metabolites. We decided to explore possibilities for such nonspecific interactions by comparing binding of our prototype inhibitor to Dnmt1 and Dnmt3a, two very similar DNA methyltransferases with closely related physiological functions.

**Fig 8:**
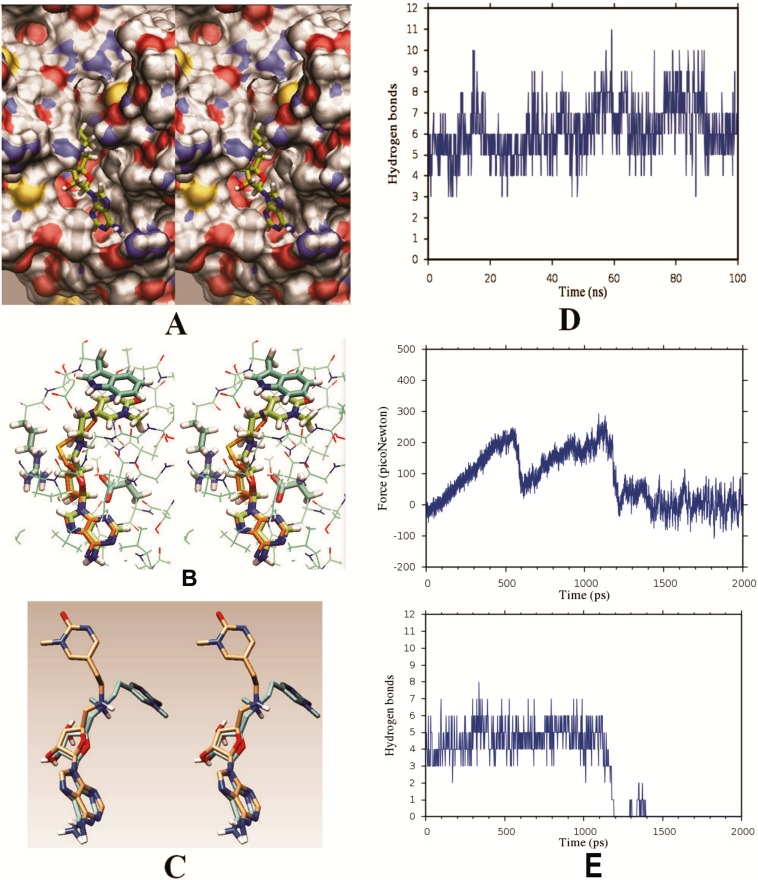
(A-E) The lead compound can bind to mouse Dnmt3a (PDB: 4U7P ref [18]). **(A)** The cross-eyed stereo image shows our prototype inhibitor bound at the AdoMet site on Dnmt3a. Similar to the complex with Dnmt1, with Dnmt3a adenine part of the inhibitor is buried in a hydrophobic pocket, while the ribose is sitting atop of a ridge formed by Glu664. Hydrogen atoms are shown in white, carbon in gray, oxygen in red, nitrogen in blue, and sulfur in yellow [41]. **(B)** Stereo image shows inhibitor (lime) bound to Dnmt3 (cyan). The adenosyl part of inhibitor closely overlaps with corresponding parts of AdoHcys in the crystal structure (orange). Similar to Dnmt1 the ribose ring on Dnmt3a is positioned between Glu664 and Arg891. The target base ring is held in its position by π-π stacking interactions with Trp893. (**C**) Stereo image shows a comparison between the extended conformation observed in the binding site of Dnmt1 (gold), and the twisted conformation observed in the binding site of Dnmt3 (cyan). (**D**) The plot shows dynamic changes in the number of binding interactions within Dnmt3a-inhibitor complex in a 100 nsec long MD simulation. With Dnmt3a our prototype inhibitor forms 6 ± 2 binding interactions, what is in average at least one interaction less than with Dnmt1. (**E**) The plots show changes in pulling force and the corresponding decrease in the number of dynamic binding interactions within Dnmt3a-inhibitor complex. The two plots were calculated using steered molecular dynamics protocols in program GROMACS [31].

We found that our prototype inhibitor can bind to human Dnmt3a (Fig 8). As expected, adenine and ribose parts on the inhibitor make similar binding interactions with Dnmt1 and Dnmt3a (Fig 8A–8C). Significant differences can be observed in binding interactions at the linker and the target base ring parts (Fig 8C). With Dnmt3a, the target base ring forms π-π stacking interactions with Trp893 (Fig 7B), and the linker is twisted deep into the AdoMet binding cavity (Fig 7C). Molecular dynamic simulations showed that Dnmt3a forms in average 6 ± 2 binding interactions with the inhibitor, which is at least one less binding interaction than with Dnmt1 (Figs 4 vs. 8D). Consequently, the pulling force for detachment of the inhibitor from Dnmt3a is 70 picoNewtons lower than for Dnmt1 (Figs 4 vs. 8E). The simulations also showed that π-π stacking interactions between the target base and Trp893 can be readily broken.

The weaker interactions between Dnmt3a and the inhibitor could possibly provide a satisfactory specificity for Dnmt1 in cells. However, if necessary we can also exploit the observed differences in binding interactions to design even more specific compounds. For example, we quickly found that a linker with *entgegen* double bond between carbon 1–2 and carbon 3–4 can bind very well to Dnmt1 but not to Dnmt3a (S3 Table, compound 42). The linker with a double bond can take only extended conformations that are complementary to the extended binding site on Dnmt1 but not to the curved binding site on Dnmt3a (Fig 8C and S3 Table).

In conclusion, apart from the mechanism-based suicide-inhibition specificity for Dnmt1 can also be achieved by carefully chosen modifications in different parts of the inhibitor.

## Conclusions and future directions

Our *in silico* studies show that it is possible to prepare a whole family of mechanism-based suicide-inhibitors that can bind to Dnmt1 as transition-state analogues. Different modifications in the lead compound structure can be used to facilitate synthesis of the inhibitor or to prepare a whole family of mechanism-based inhibitors with different pharmacological properties. Many of the presented compounds can be prepared for less than 5000 USD in material costs in a total of 4 to 6 synthetic steps [26, 27]. Briefly, 1-methyl-pyrimidin-2-one and its different modifications can be purchased commercially or prepared by Biginelli reaction from methyl-urea and different dialdehydes [26, 27]. The prepared target base can be attached to different linkers using Suzuki addition [26, 27]. The linker has to be fully prepared before the addition to avoid cross-reactivity with the target base. The prepared target base with a linker can be attached by substitution to commercially available adenosyl derivatives, or some of its in-house prepared modifications [26, 27].

The presented *in silico* approaches can also be extended in the future to prepare compounds specific for Dnmt1, Dnmt2, Dnmt3, or some bacterial DNA methyltransferases [34–36]. Selective compounds can be prepared by exploiting subtle differences between different DNA methyltransferase in their active site and interaction with the cofactor [20]. The hope is to design inhibitors that will have a preference for different DNA methylation processes in cells [6].

## Materials and methodology

All calculations have been performed on Bullx DLC 720 high-performance computer at the Center for Advanced Computing and Modeling at the University of Rijeka (http://cnrm.uniri.hr/). A typical calculation used 50–100 nodes that were integrated by Infiniband FDR 54 Gbps connections. Each node contains two Intel Xeon E5-2690 v3 processors, NVIDIA K40 GPU and 64GB RAM. All simulations were prepared at NVIDIA CUDA Teaching center at the University of Rijeka.

### Rigid body docking and analysis

The initial “rigid-body-docking’ calculations used programs Autodock Vina as a plug-in application in UCSF-Chimera [37, 38]. The protein coordinates were acquired from Protein data bank, while the ligand structures were drawn in ChemAxon Marvin [39]. Prior to docking, the ligand structures were tested with several programs to make sure that conversion between file formats did not distort chiral centers or aromatic bonds. In the first step, both the protein and the ligand were protonated at pH = 7.2 using Amber99SB force-field tool in Chimera program. Then, the search volume box 25.0 X 23.5 X 19.0 Å was prepared, which included AdoHcy binding site, the active site where the target cytosine binds, and the interface between the catalytic domains [18]. The crystal water molecules have been deleted, except for the molecules that had hydrogen bonds with the residues at the AdoMet sites. In the latter case, we did docking with and without the water molecules. Docking was judged as successful if at least one of the nine conformers showed overlap with adenine rings and the target cytosine in the corresponding crystal structure (Fig 3).

### Molecular dynamics simulations and analysis

Classical molecular mechanics and molecular dynamics (MM/MMD) simulations were calculated using program package GROMACS [30, 31]. The protein was processed with pdb2gmx, while the ligand was processed with ACPYPE10 [40]. ACPYPE is an interface for Antechamber (part of AmberTools11) which is used to generate different topology types for various force fields (CHARMM, GROMOS, AMBER, OPLS) [40]. Amber99SB force field was used for proteins, while GAFF was used for nonstandard residues (i.e. ligands). Following the parameterization, all coordinate files were merged into a single coordinate file, and all topology files into single topology file.

We used rhombic dodecahedron solvent box, type and TIP3P model for water molecules, and 150 mM NaCl plus additional Na+ ions that were required for neutralization. The prepared system was minimized using a combination of steepest descent and conjugate gradient algorithms. When the most stable state was achieved the temperature was introduced and the system was equilibrated to 310 K (NVT equilibration, V-rescale). Next, the pressure was equilibrated to 1 atm (NPT equilibration, Parrinello-Rahman). No restraints were used for the protein or the ligand when the system was minimized, but in NPT and NVT equilibration protein and ligand were restrained. The outputs of minimization and equilibration MD simulations provide insight into the potential energy of the system based on minimization of the temperature in NVT simulation, and minimization of the pressure in NPT simulation.

We calculated MD simulations for entire mammalian Dnmt1 in total duration of 20–100 ns, 10–50 million steps, 2 fs step-time, with a total of 100–2000 recorded frames. Following MM/MD simulations RMSD plots, “Cys 1226 and carbon 6 distance” plots, and H-bond plots were calculated using built-in GROMACS and VMD utilities [30, 31, 41].

### Steered molecular dynamics

Steered molecular dynamics simulation used GROMACS suite with the same set-up parameters as the rest of molecular dynamics simulations [30, 31]. The inhibitor or the active site loop were pulled from the center of mass away from the center of mass of the protein at 0.001 nm/ps with harmonic force constant of 1000 kJ mol^-1^ nm^-2^. The system was solvated in an octahedron box with faces on 5 nm distance from the protein. TIP3P model was used for water molecules, salt concentration was set to 0.15 mol/dm3 by adding Na+ and Cl- ions. The systems were minimized with steepest descent algorithm and then equilibrated in 500 thousand steps at a temperature of 310 K and a pressure of 1 bar. Steered molecular dynamics were carried out in 1 million steps in 2 ns with coordinates, forces and velocities logged every 10 ps.

### QM and QM/MM simulations

Frontier molecular orbitals were calculated using program US-GAMESS [42]. The molecules were drawn in Avogadro [43], the calculations were set-up using “SCFTYP = RHF RUNTYP = ENERGY DFTTYP = B3LYP” protocols with 6-31G basis set, water medium, and initial MO guess set as Hückel. The resulting molecular orbitals and the calculated energies have been visualized using VMD [41].

CP2K program was used for hybrid quantum mechanics and molecular mechanics (QM/MM) simulation. QM simulations used density functional theory (DFT) that was based on hybrid Gaussian and plane waves density functional [44]. TZV2P-GTH basis sets and B3LYP exchange-correlation energy functional were used for 19 QM atoms in the active site. CHARMM force field parameters were used for MM atoms. Force field parameters for ligand were generated by Acpype [40]. PSF topology used in CP2K is generated by VMD using automatic PSF builder plugin [41].

Nudged elastic band method was used for transition state search [45, 46]. PyMOL is used for selection of QM atoms in QM/MM simulation, and PyMOL plug-in was used for generation of QMMM section in CP2K input file. Replicas used in the nudged elastic band were drawn in Avogadro [43].

## Supporting information

S1 Table Modifications in the adenine ring. (DOC)

S2 Table Modifications in the ribose ring. (DOC)

S3 Table Modifications in the linker. (DOC)

S4 Table Modifications in the target base ring. (DOC)

S5 Table Decrease in the inhibitor flexibility by modifications at multiple sites. (DOC)

S1 Movie Dynamic changes in binding interactions within Dnmt1-inhibitor complex (PDB: 4DA4 [18]). Molecular dynamics simulations were used to visualize dynamic changes in binding interactions that are not readily visible in static crystal structures [30, 31]. The inhibitor and the interacting amino acids are shown in licorice, while the rest of the protein is shown as a ribbon. The gold dashed lines show binding interactions between inhibitor and binding site residues. For clarity, the surrounding solvent molecules are not shown. The MD simulations show that the target base ring is held in the active site cavity primarily by an ion-dipole interaction between its carbonyl oxygen and Arg 1312 and Arg1310. The active site Cys1226 is hovering above the target base ring within van der Waals contact. Mobility and position of the target base ring in the active site cavity depends on length and flexibility of the linker. The adenine ring is buried behind Met1169, where its nitrogen atoms form several binding interactions with Asp 1190 and Cys 1191. The ribose is anchored to Glu1168 with two hydrogen bonds that are directed towards H atoms on the two OH groups. The oxygen atoms on the two OH groups can make dynamic binding interactions with Arg1573, which can be stabilized in its binding position by π-π stacking with Trp1170.
(AVI)

S2 Movie Mobility of the inhibitor within the active site in Dnmt1-inhibiotr complex (PDB: 4DA4 [18]). Steered molecular dynamics was used to compare different binding interactions within Dnmt1-inhibitor complex. The simulation also illustrates dynamics processes that guide association and dissociation of the inhibitor. In this protocol, the center of mass of the inhibitor is gradually pulled out from the binding site cavity by a force that is constantly adjusted to the opposing forces of binding interactions [31]. We find that the inhibitor is displaced from its binding site in several steps. The target base ring and the linker are displaced first as soon as the active site loop is released. Surprisingly, the ribose and the adenine rings remain bound to the cofactor site even when the target base ring and the linker are completely dissociated. The ribose dissociates when its binding interactions with Glu1168 and Arg1573 are broken. The adenosine ring is last to dissociate as it slips from its hydrophobic cavity.
(AVI)

S3 Movie Formation of a covalent bond between our lead compound and the active site cysteine 1226 (prepared in VMD [41]). QM/MM simulation show how nucleophilic orbitals on the sulfur atom can come in the same plane as electrophilic orbitals on carbon 6 on the target base. Hydrogen atoms are shown in white, carbon in cyan, oxygen in red, nitrogen in blue, and sulfur in yellow. The first panel shows one of the GROMACS MM/MD simulation frames that was used as a starting point for nudged elastic band QM/MM studies [44]. The active site Cys 1226 is directly above the plane of the target base ring 3.8 Å from the carbon 6. Both Cys 1226 and carbon 6 on the target base are protonated. The reaction starts when the active site cysteine is deprotonated with an OH^-^ ion. OH^-^ ions are known to penetrate in the active site [19, 20, 29]. Next, the newly formed Cys 1226 anion collides with a hydrogen atom at the carbon 6 on the target base. The collision can drive the HOMO orbitals on nucleophilic sulfur anion in the same plane as LUMO orbitals on the carbon 6 on the target base. The transition state intermediate forms when the carbon 6 is deprotonated by the second OH^-^ ion (deprotonation of the carbon 6 takes place below the ring so it is not fully visible in the presented angle). In the final step, the covalent adduct is formed, and the new bonds are minimized to achieve the optimal bond angles. In sum, our MM/MD and QM/MM simulations show that the suicide-inhibition depends on two sets of conformational changes. First, the closing of the active site loop has to drive the nucleophilic Cys1226 into a tight Van der Waals contact with carbon 6 on the target base. Second, the correct angle between reactive atoms has to be achieved by rotation of the target base ring around the axis formed by hydrogen bonds with Arg 1310 and Arg 1312.
(AVI)

S4 Movie Inhibitor binding to different conformations in the active site of Dnmt1 (prepared in VMD [41]). We used steered molecular dynamic to analyze how stability of Dnmt1-inhibitor complex can be affected by the active site loop (amino acids 1220 to 1236). The simulation is started with the active site loop in its closed position, which represents fully formed a catalytic complex (PDB: 4DA4 [18]). During the simulation, center of mass of the active site loop is gradually pulled out to its open position using force that is proportional to the opposing forces created by the dynamic changes in the loop structure. We found that the opening of the loop does not lead to immediate dissociation of the inhibitor. Rather, gradual opening of the loop leads to increase in distance between the target base and Cys1226, and thus decrease in chances for the formation of a covalent adduct and the suicide-inhibition.
(AVI)

## References

[1] Covic M, Karaca E, Lie D. Epigenetic regulation of neurogenesis in the adult hippocampus. Heredity. 2010;105(1):122–34. 10.1038/hdy.2010.27 20332807

[2] Romanoski CE, Glass CK, Stunnenberg HG, Wilson L, Almouzni G. Epigenomics: Roadmap for regulation. Nature. 2015;518(7539):314–6. 10.1038/518314a 25693562

[3] Pennarossa G, Maffei S, Campagnol M, Tarantini L, Gandolfi F, Brevini TA. Brief demethylation step allows the conversion of adult human skin fibroblasts into insulin-secreting cells. Proceedings of the National Academy of Sciences. 2013;110(22):8948–53. 10.1073/pnas.1220637110 PMC3670366 23696663

[4] Khavari DA, Sen GL, Rinn JL. DNA methylation and epigenetic control of cellular differentiation. Cell Cycle. 2010;9(19):3880–3. 20890116 10.4161/cc.9.19.13385

[5] Sen GL, Reuter JA, Webster DE, Zhu L, Khavari PA. DNMT1 maintains progenitor function in self-renewing somatic tissue. Nature. 2010;463(7280):563–7. 10.1038/nature08683 20081831 PMC3050546

[6] Svedruzic ZM. Dnmt1 structure and function. Prog Mol Biol Transl Sci. 2011;101:221–54. 10.1016/B978-0-12-387685-0.00006-8 21507353

[7] Svedružić ZM, Popovic K, Sendula-Jengic V. Decrease in catalytic capacity of γ-secretase can facilitate pathogenesis in sporadic and Familial Alzheimer's disease. submitted. 2015. 10.1016/j.mcn.2015.06.002 26051801

[8] Svedružić ZM, Popovic K, Smoljan I, Sendula-Jengic V. Modulation of gamma-Secretase Activity by Multiple Enzyme-Substrate Interactions: Implications in Pathogenesis of Alzheimer's Disease. PloS one. 2012;7(3):e32293 10.1371/journal.pone.0032293 22479317 PMC3316526

[9] Babenko O, Kovalchuk I, Metz GA. Epigenetic programming of neurodegenerative diseases by an adverse environment. Brain research. 2012;1444:96–111. 10.1016/j.brainres.2012.01.038 22330722

[10] Gros C, Fahy J, Halby L, Dufau I, Erdmann A, Gregoire J-M, et al DNA methylation inhibitors in cancer: recent and future approaches. Biochimie. 2012;94(11):2280–96. 10.1016/j.biochi.2012.07.025 22967704

[11] Heerboth S, Lapinska K, Snyder N, Leary M, Rollinson S, Sarkar S. Use of epigenetic drugs in disease: an overview. Genetics & epigenetics. 2014;6:9. 25512710 10.4137/GEG.S12270 PMC4251063

[12] Svedruzic ZM. Mammalian cytosine DNA methyltransferase Dnmt1: enzymatic mechanism, novel mechanism-based inhibitors, and RNA-directed DNA methylation. Curr Med Chem. 2008;15(1):92–106. 18220765 10.2174/092986708783330700

[13] Xu F, Mao C, Ding Y, Rui C, Wu L, Shi A, et al Molecular and enzymatic profiles of mammalian DNA methyltransferases: structures and targets for drugs. Current medicinal chemistry. 2010;17(33):4052 20939822 10.2174/092986710793205372 PMC3003592

[14] Qin W, Leonhardt H, Pichler G. Regulation of DNA methyltransferase 1 by interactions and modifications. Nucleus (Austin, Tex). 2011;2(5):392–402. Epub 2011/10/13. 10.4161/nucl.2.5.17928 21989236

[15] Di Ruscio A, Ebralidze AK, Benoukraf T, Amabile G, Goff LA, Terragni J, et al DNMT1-interacting RNAs block gene-specific DNA methylation. Nature. 2013;503(7476):371–6. 10.1038/nature12598 24107992 PMC3870304

[16] Fersht A. Structure and Mechanism in Protein Science: A Guide to Enzyme Catalysis and Protein Folding (Hardcover). 1st ed: W. H. Freeman; 1st edition 1998 650 p.

[17] Wu JC, Santi DV. Kinetic and catalytic mechanism of HhaI methyltransferase. The Journal of biological chemistry. 1987;262:4778–86. 3558369

[18] Song J, Teplova M, Ishibe-Murakami S, Patel DJ. Structure-based mechanistic insights into DNMT1-mediated maintenance DNA methylation. Science (New York, NY). 2012;335(6069):709–12. Epub 2012/02/11. 10.1126/science.1214453 PMC4693633 22323818

[19] Svedruzic ZM, Reich NO. The mechanism of target base attack in DNA cytosine carbon 5 methylation. Biochem. 2004;43(36):11460–73. 15350132 10.1021/bi0496743

[20] Svedruzic ZM, Reich NO. DNA cytosine C5 methyltransferase Dnmt1: catalysis-dependent release of allosteric inhibition. Biochem. 2005;44(27):9472–85. 15996102 10.1021/bi050295z

[21] Song J, Rechkoblit O, Bestor TH, Patel DJ. Structure of DNMT1-DNA complex reveals a role for autoinhibition in maintenance DNA methylation. Science (New York, NY). 2011;331(6020):1036–40. Epub 2010/12/18. 10.1126/science.1195380 PMC4689315 21163962

[22] Svedruzic ZM, Reich NO. Mechanism of allosteric regulation of Dnmt1's processivity. Biochem. 2005;44(45):14977–88. 16274244 10.1021/bi050988f

[23] Estabrook RA, Nguyen TT, Fera N, Reich NO. Coupling sequence-specific recognition to DNA modification. The Journal of biological chemistry. 2009;284(34):22690–6. 10.1074/jbc.M109.015966 19497854 PMC2755677

[24] Medina-Franco JL, Caulfield T. Advances in the computational development of DNA methyltransferase inhibitors. Drug discovery today. 2011;16(9):418–25. 21315180 10.1016/j.drudis.2011.02.003

[25] Nikolić P, Miletić V, Odorčić I, Svedružić ŽM. Insilico optimization of the first DNA-independent mechanism-based inhibitor of the mammalian DNA methyltransferase Dnmt1 In: Medina-Franco J, editor. EPI-INFORMATICS Discovery And Development Of Small Molecule Epigenetic Drugs And Probes: Elsevier, Academic Press; 2016 p. 440.

[26] Merino P. Chemical Synthesis of Nucleoside Analogues: John Wiley & Sons; 2013.

[27] Vorbrüggen H, Ruh-Pohlenz C. Handbook of Nucleoside Synthesis: Wiley; 2001.

[28] Daigle SR, Olhava EJ, Therkelsen CA, Basavapathruni A, Jin L, Boriack-Sjodin PA, et al Potent inhibition of DOT1L as treatment of MLL-fusion leukemia. Blood. 2013;122(6):1017–25. Epub 2013/06/27. 10.1182/blood-2013-04-497644 23801631 PMC3739029

[29] Yang J, Lior-Hoffmann L, Wang S, Zhang Y, Broyde S. DNA cytosine methylation: structural and thermodynamic characterization of the epigenetic marking mechanism. Biochemistry. 2013;52(16):2828–38. Epub 2013/03/27. 10.1021/bi400163k 23528166 PMC3687104

[30] Hess B, Kutzner C, van der Spoel D, Lindahl E. GROMACS 4: Algorithms for Highly Efficient, Load-Balanced, and Scalable Molecular Simulation. J Chem Theory Comput. 2008;4:435–47. 10.1021/ct700301q 26620784

[31] Van Der Spoel D, Lindahl E, Hess B, Groenhof G, Mark AE, Berendsen HJ. GROMACS: fast, flexible, and free. Journal of computational chemistry. 2005;26(16):1701–18. Epub 2005/10/08. 16211538 10.1002/jcc.20291

[32] Takeshita K, Suetake I, Yamashita E, Suga M, Narita H, Nakagawa A, et al Structural insight into maintenance methylation by mouse DNA methyltransferase 1 (Dnmt1). Proceedings of the National Academy of Sciences of the United States of America. 2011;108(22):9055–9. Epub 2011/04/27. 10.1073/pnas.1019629108 21518897 PMC3107267

[33] Isakovic L, Saavedra OM, Llewellyn DB, Claridge S, Zhan L, Bernstein N, et al Constrained (l-)-S-adenosyl-l-homocysteine (SAH) analogues as DNA methyltransferase inhibitors. Bioorganic & medicinal chemistry letters. 2009;19(10):2742–6. 19364644 10.1016/j.bmcl.2009.03.132

[34] Zhou L, Cheng X, Connolly BA, Dickman MJ, Hurd PJ, Hornby DP. Zebularine: a novel DNA methylation inhibitor that forms a covalent complex with DNA methyltransferases. J Mol Biol. 2002;321(4):591–9. 10.1016/S0022-2836(02)00676-9 12206775 PMC2713825

[35] Li S, Du J, Yang H, Yin J, Ding J, Zhong J. Functional and structural characterization of DNMT2 from Spodoptera frugiperda. Journal of molecular cell biology. 2013;5(1):64–6. Epub 2012/10/30. 10.1093/jmcb/mjs057 23103599

[36] Guo X, Wang L, Li J, Ding Z, Xiao J, Yin X, et al Structural insight into autoinhibition and histone H3-induced activation of DNMT3A. Nature. 2015;517(7536):640–4. Epub 2014/11/11. 10.1038/nature13899 25383530

[37] Pettersen EF, Goddard TD, Huang CC, Couch GS, Greenblatt DM, Meng EC, et al UCSF Chimera—a visualization system for exploratory research and analysis vesrion Journal of computational chemistry. 2004;25(13):1605–12. Epub 2004/07/21. 15264254 10.1002/jcc.20084

[38] Trott O, Olson AJ. AutoDock Vina: improving the speed and accuracy of docking with a new scoring function, efficient optimization, and multithreading. Journal of computational chemistry. 2010;31(2):455–61. Epub 2009/06/06. 10.1002/jcc.21334 19499576 PMC3041641

[39] ChemAxon. Marvin was used for drawing, displaying and characterizing chemical structures, substructures and reactions, Marvin 15.0.1 (version number), 2015, ChemAxon http://www.chemaxon.com.

[40] Sousa da Silva AW, Vranken WF. ACPYPE—AnteChamber PYthon Parser interfacE. BMC research notes. 2012;5:367 Epub 2012/07/25. 10.1186/1756-0500-5-367 22824207 PMC3461484

[41] Humphrey W, Dalke A, Schulten K. VMD—Visual Molecular Dynamics. J Mol Graph. 1996;14(version 1.9.2):33–8. 8744570 10.1016/0263-7855(96)00018-5

[42] Gordon MS, Schmidt MW. Advances in electronic structure theory: GAMESS a decade later. Theory and Applications of Computational Chemistry: the first forty years. 2005:1167–89.

[43] Hanwell MD, Curtis DE, Lonie DC, Vandermeersch T, Zurek E, Hutchison GR. Avogadro: an advanced semantic chemical editor, visualization, and analysis platform. Journal of cheminformatics. 2012;4(1):17 Epub 2012/08/15. 10.1186/1758-2946-4-17 22889332 PMC3542060

[44] VandeVondele J, Krack M, Mohamed F, Parrinello M, Chassaing T, Hutter J. Quickstep: Fast and accurate density functional calculations using a mixed Gaussian and plane waves approach. Computer Physics Communications. 2005;167(2):103–28

[45] Henkelman G, Jónsson H. Improved tangent estimate in the nudged elastic band method for finding minimum energy paths and saddle points. The Journal of chemical physics. 2000;113(22):9978–85

[46] Henkelman G, Uberuaga BP, Jónsson H. A climbing image nudged elastic band method for finding saddle points and minimum energy paths. The Journal of chemical physics. 2000;113(22):9901–4.

