# Supplementary material for: In silico design of the first DNA-independent mechanism-based inhibitor of mammalian DNA methyltransferase Dnmt1
Source: PLoS One. 2017 Apr 11;12(4):e0174410. doi: 10.1371/journal.pone.0174410 (PMC5388339; doi:10.1371/journal.pone.0174410)
Supplement: S1 Table — (DOC) [file pone.0174410.s001.doc]

In all tables, **the first column** shows the lead compound or its different modifications labeled with corresponding numbers. The LogD values for each modification were calculated using the weighted option in ChemAxon Marvin 16.12.5.0 . The LogD value for the lead compound is -2.67, thus any modification that has a LogD value higher than -2.67 represents a favorable LogD modification. **The second column** shows “Cys1226-carbon-6” distance plots. The plots can be used to evaluate likelihood for a mechanism-based suicide-inhibition. A distance below 4 Å indicates that active site Cys1226 and the target base are in a close Van der Waals contact that can support the formation of the covalent adduct and a mechanism-based suicide-inhibition (Fig 6). The **third column** shows RMSD values relative to the initial complex that was prepared using a rigid body docking protocol . Different binding orientations can be seen as large peaks or steps in plots of “average-ligand-RMSD” values. Distinct steps in RMSD plots represent distinct conformations of the ligand within the complex, while uniform RMSD plots with low variability represent a tightly bound inhibitor. All MM/MD simulations started with 20 nsec simulations. For more detailed description we used 100 nsec simulations. The modifications that give favorable LogD values and “Cys1226-carbon-6” distance plots are marked with red numbers.

**Table 1A. Modifications at the C6 position in adenine ring:**

| **Structure modification and its number.**  **LogD @pH=7.4** | **GROMACS MM/MD frames**  **Cys1226 ringC6 distance** | **GROMACS MM/MD frames**  **RMSD Ligand**  **relative to the first frame** |
| --- | --- | --- |
| 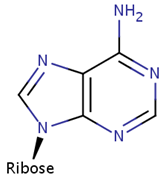  Lead-compound as a reference  Log(D)=-2.67 | 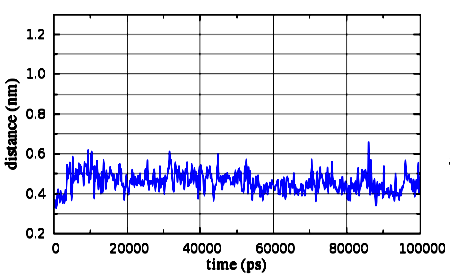 | 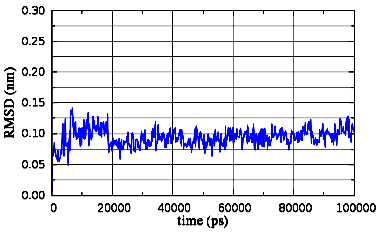 |
| 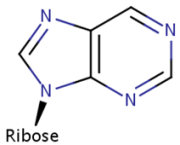  **1**  Log(D)=-2.84 | With this modification, the inhibitor cannot simultaneously bind in the active site and AdoMet site and act as a transition state analogue (Fig 3). | |
| 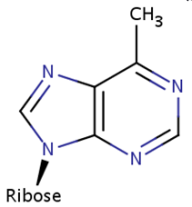  **2**  Log(D)=-2.60 | **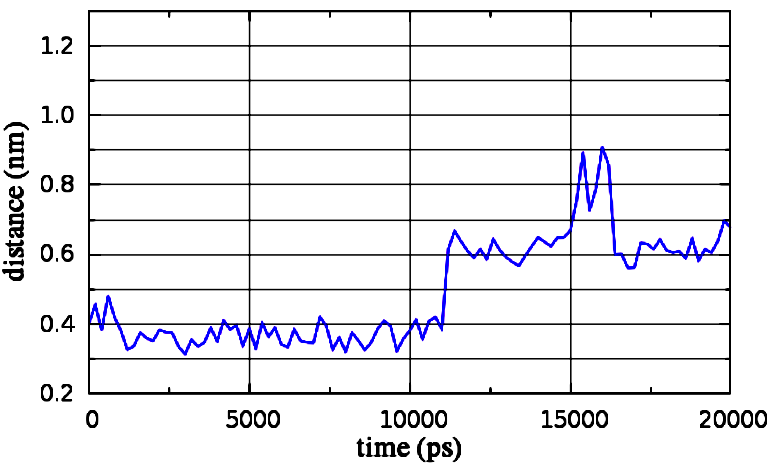** | 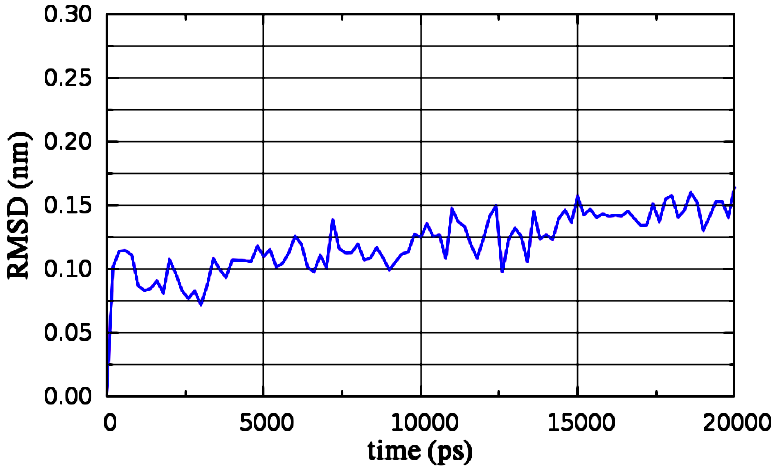 |
| 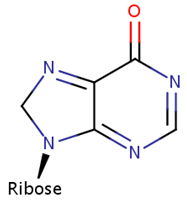  **3**  Log(D)=-2.44 | 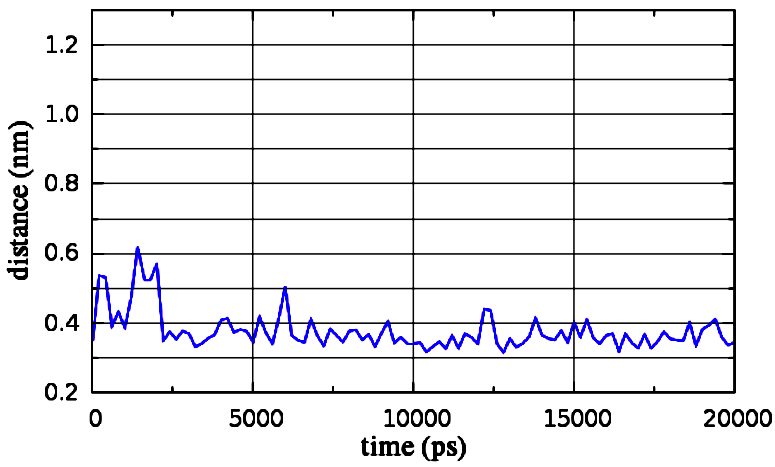 | 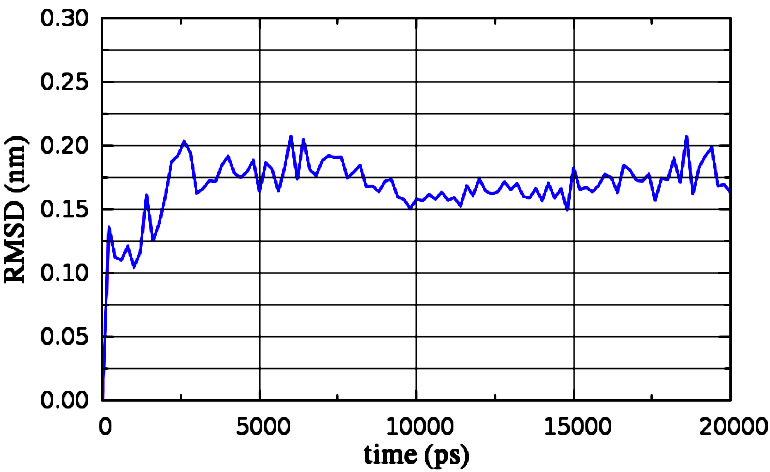 |

**Table 1B. Modifications in hetero atoms in adenosine ring:**

| **Structure modification and its number.**  **LogD @pH=7.4** | **GROMACS MM/MD frames**  **Cys1226 ringC6 distance** | **GROMACS MM/MD frames**  **RMSD Ligand**  **relative to the first frame** |
| --- | --- | --- |
| 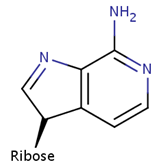**4**  Log(D)=-2.64 | With this modification the inhibitor cannot simultaneously bind to the active site and AdoMet site and act as a transition state analogue (Fig 3). | |
| 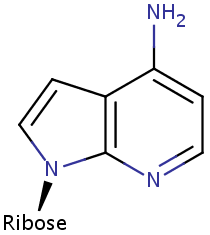**5**  Log(D)=-2.84 | 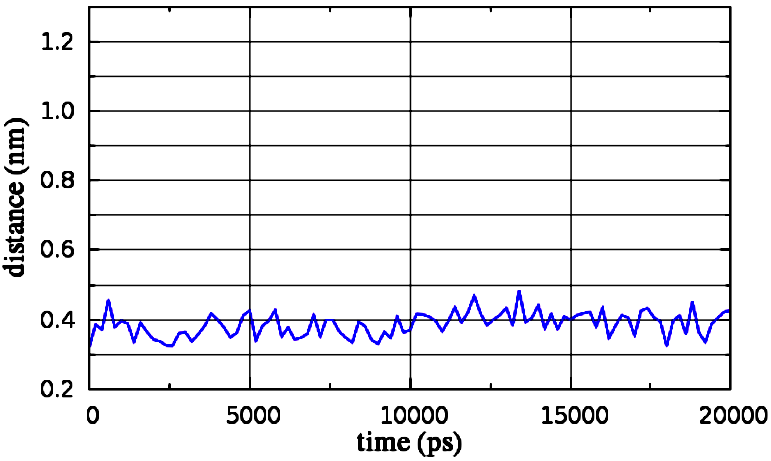 | 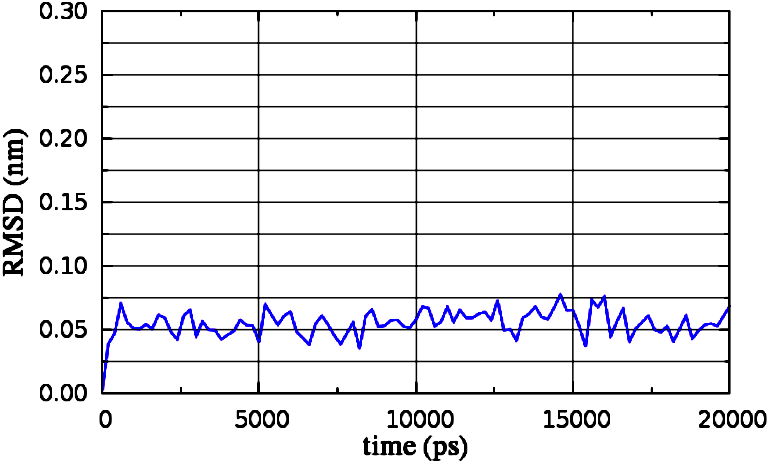 |
| 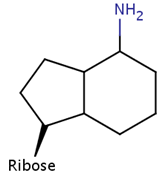  **6**  Log(D)=-1.2 | **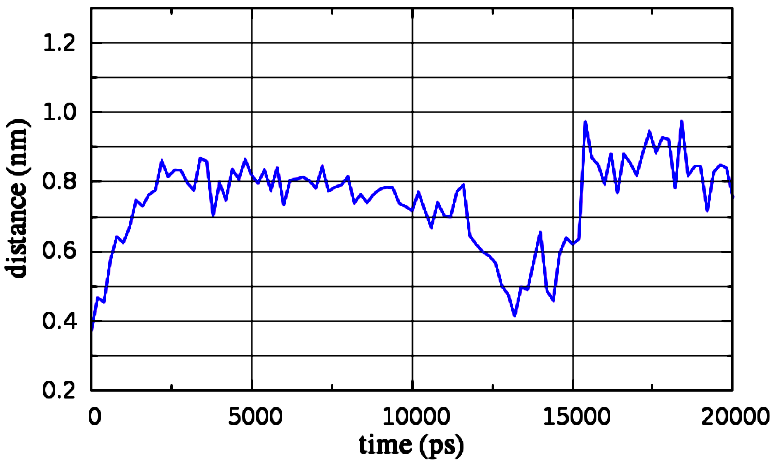** | 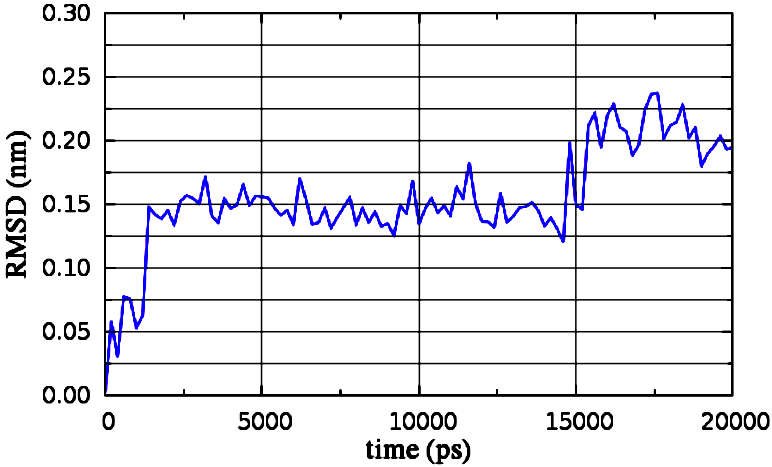 |
| 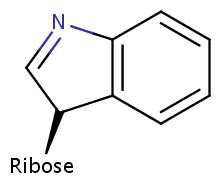**7**  Log(D)=-1,31 | **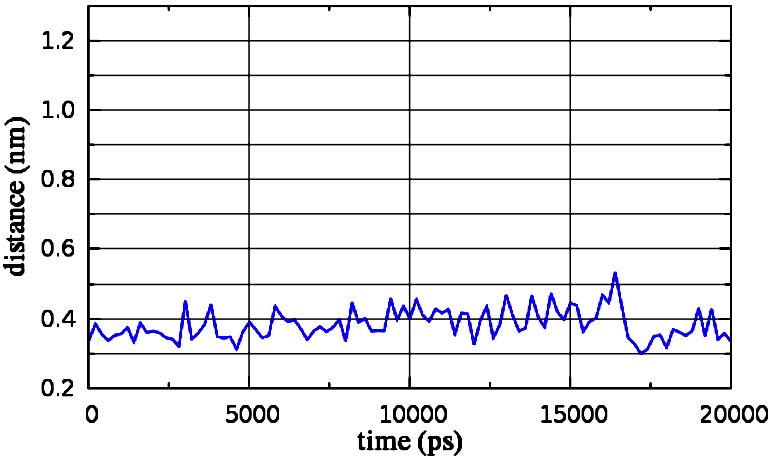** | 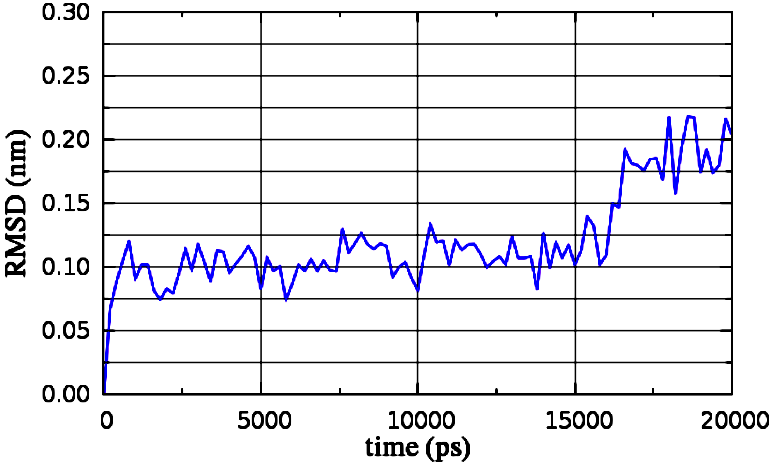 |
