# Supplementary material for: In silico design of the first DNA-independent mechanism-based inhibitor of mammalian DNA methyltransferase Dnmt1
Source: PLoS One. 2017 Apr 11;12(4):e0174410. doi: 10.1371/journal.pone.0174410 (PMC5388339; doi:10.1371/journal.pone.0174410)
Supplement: S3 Table — (DOC) [file pone.0174410.s003.doc]

In all tables, **the first column** shows the lead compound or its different modifications labeled with corresponding numbers. The LogD values for each modification were calculated using the weighted option in ChemAxon Marvin 16.12.5.0. The LogD value for the lead compound is -2.67, thus any modification that has a LogD value higher than -2.67 represents a favorable LogD modification. **The second column** shows “Cys1226-carbon-6” distance plots. The plots can be used to evaluate likelihood for a mechanism-based suicide-inhibition. A distance below 4 Å indicates that active site Cys1226 and the target base are in a close Van der Waals contact that can support the formation of the covalent adduct and a mechanism-based suicide-inhibition (Fig 6). The **third column** shows RMSD values relative to the initial complex that was prepared using a rigid body docking protocol . Different binding orientations can be seen as large peaks or steps in plots of “average-ligand-RMSD” values. Distinct steps in RMSD plots represent distinct conformations of the ligand within the complex, while uniform RMSD plots with low variability represent a tightly bound inhibitor. All MM/MD simulations started with 20 nsec simulations. For more detailed description we used 100 nsec simulations. The modifications that give favorable LogD values and “Cys1226-carbon-6” distance plots are marked with red numbers.

**Table 3A. Modifications in tail section of the linker:**

| **Structure modification and its number.**  **LogD @pH=7.4** | **GROMACS MM/MD frames**  **Cys1226 ringC6 distance** | **GROMACS MM/MD frames**  **RMSD Ligand**  **relative to the first frame** |
| --- | --- | --- |
| 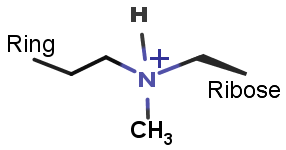  Protonated lead compound as reference  Log(D)=-2.67 | 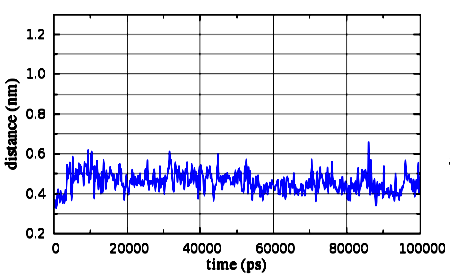 | 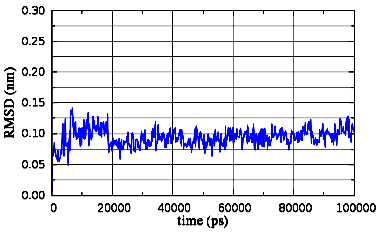 |
| 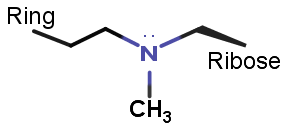  -1.88  Lead compound as reference  Log(D)=-2.67 | 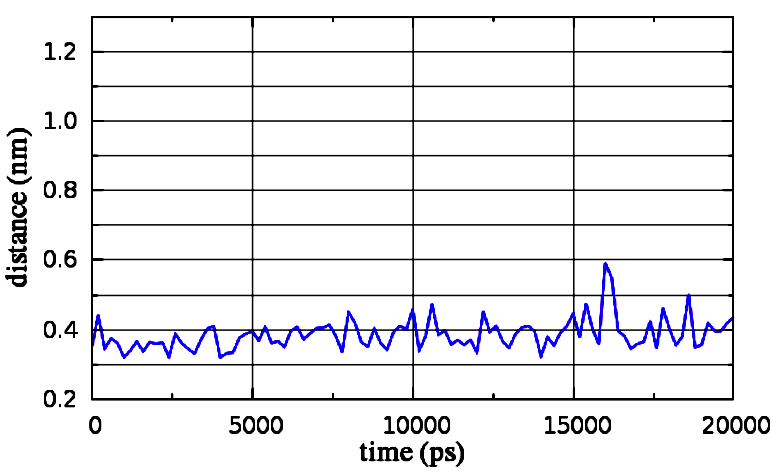 | 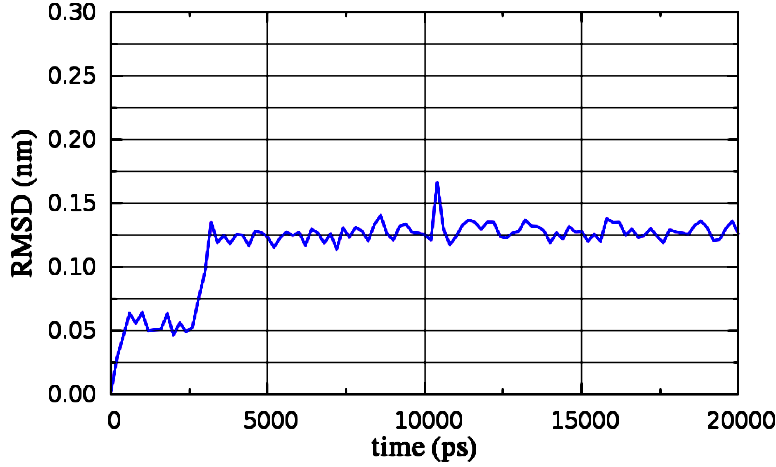 |
| 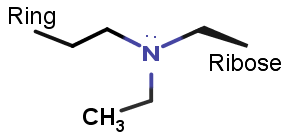  **30**  Log(D)=-1.34 | **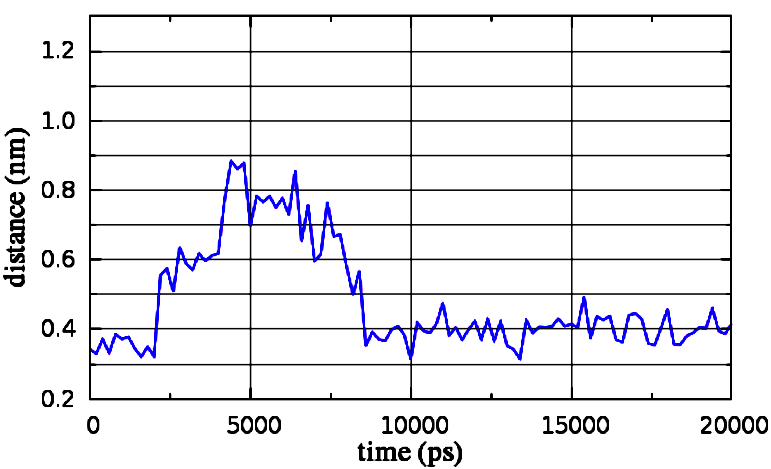** | 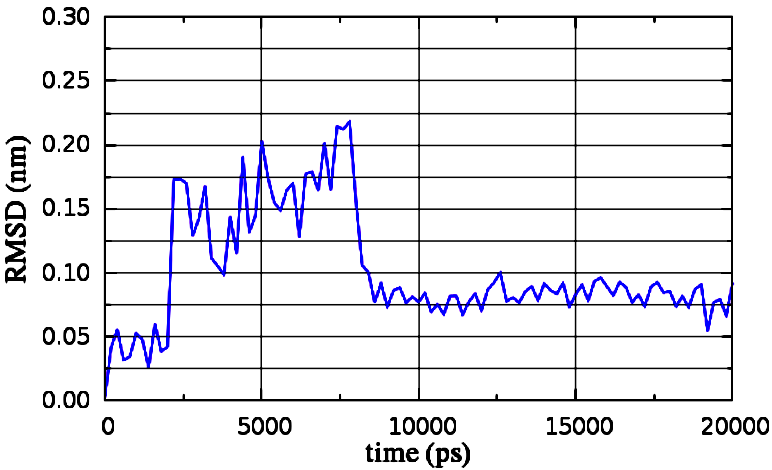 |

With these modifications the inhibitor cannot bind simultaneously to the active site and AdoMet site and act as a transition state analogue (Fig 3).

| 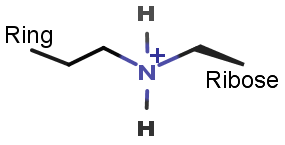  **31**  Log(D)=-2.17 | 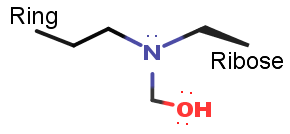  **32**  Log(D)=-2.67 | 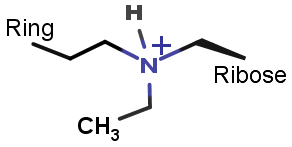  **33**  Log(D)=-1.34 |
| --- | --- | --- |
| 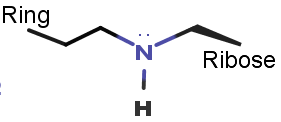  **34**  Log(D)=-2.17 | 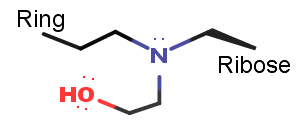  **35**  Log(D)=-2.08 |  |

**Table 3B. Modifications in flexibility of the linker:**

| **Structure modification and its number.**  **LogD @pH=7.4** | **GROMACS MM/MD frames**  **Cys1226 ringC6 distance** | **GROMACS MM/MD frames**  **RMSD Ligand**  **relative to the first frame** |
| --- | --- | --- |
| 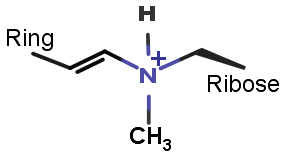  **36**  Log(D)=-2.16 | **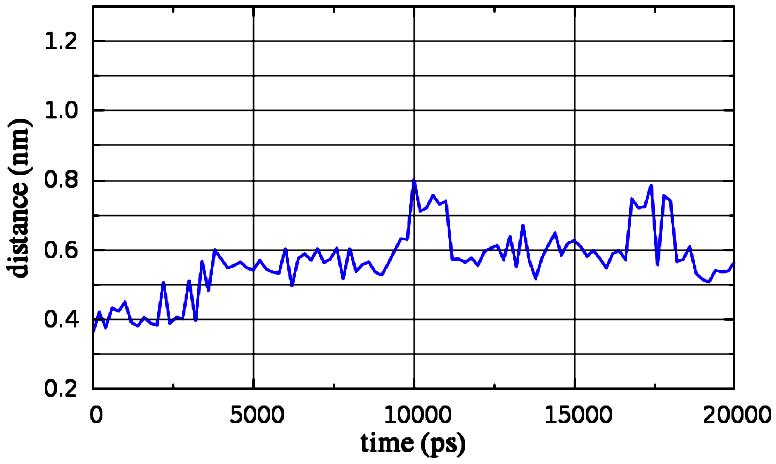** | 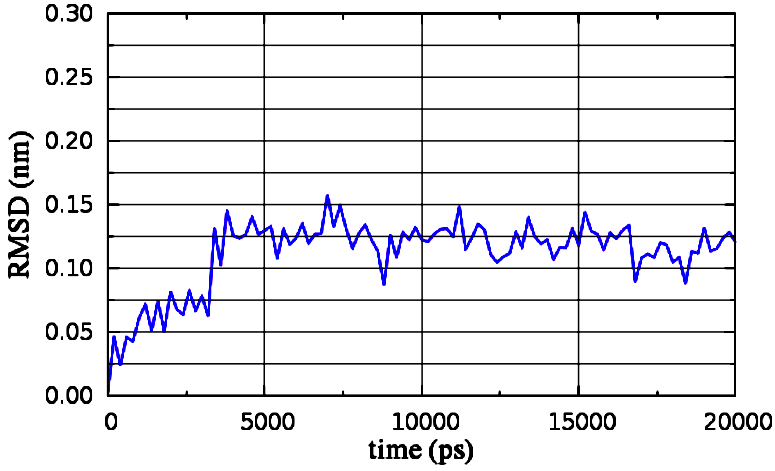 |
| 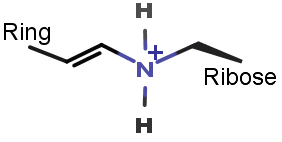  **37**  Log(D)=-2.41 | **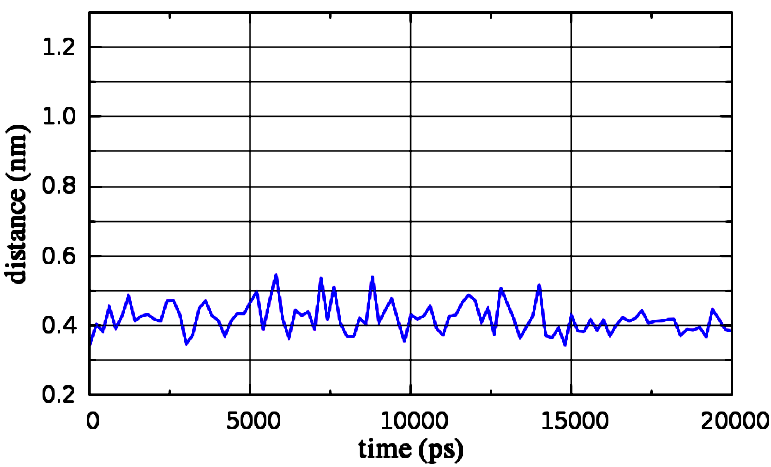** | 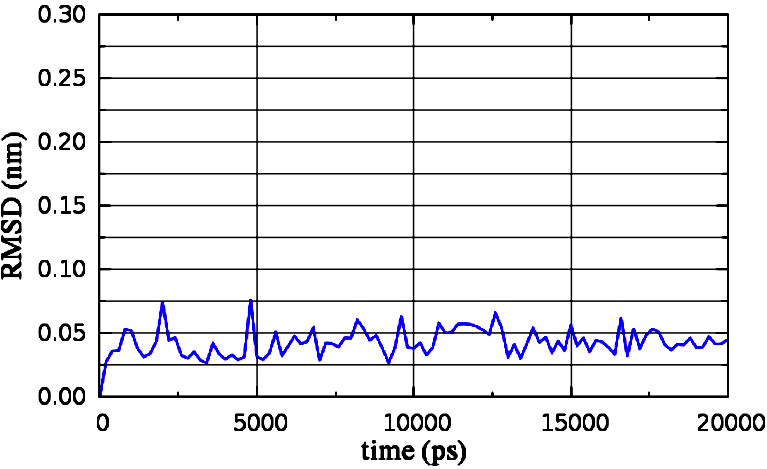 |
| 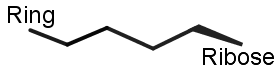  **38**  Log(D)=1.44 | 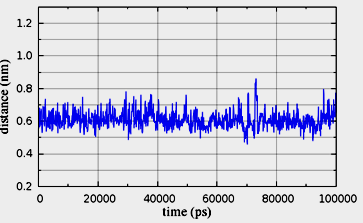 | 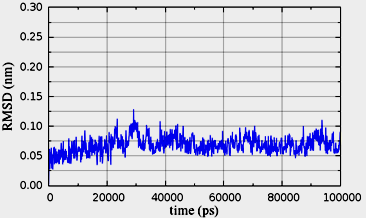 |
| 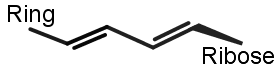  **39**  Log(D)=0.87 | 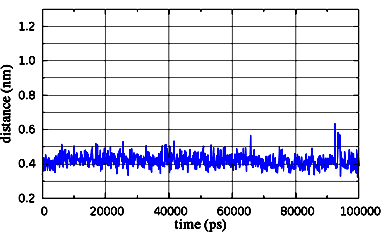 | 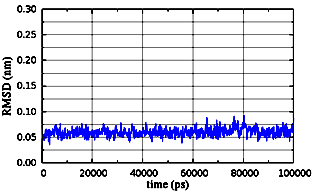 |
| 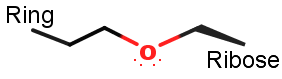  **40**  Log(D)=0.06 | 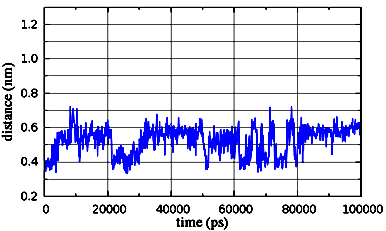 | 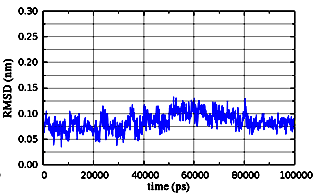 |
| 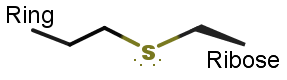  **41**  Log(D)=-1.21 | 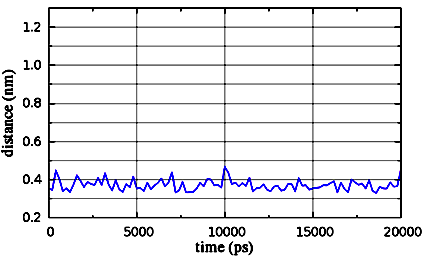 | 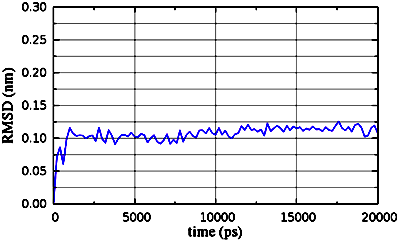 |
| 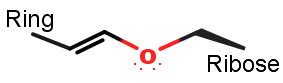  **42**  Log(D)=0.07 | 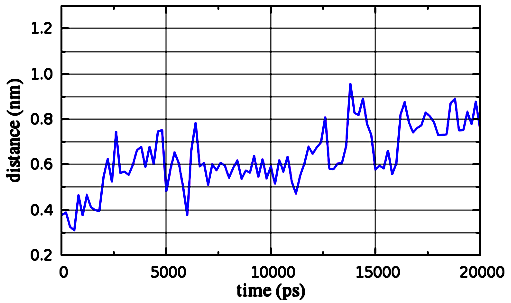 | 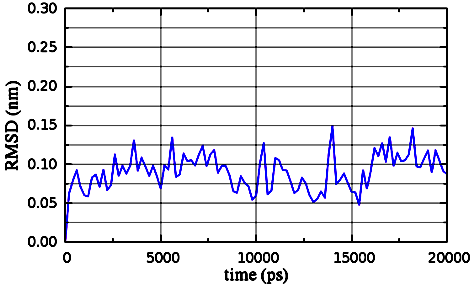 |
| 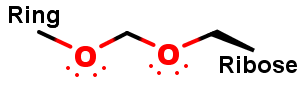  **43**  Log(D)=-2.07 | 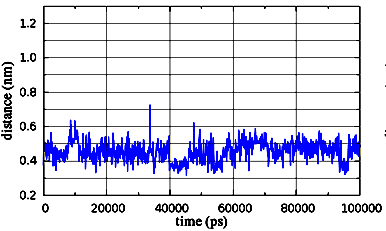 | 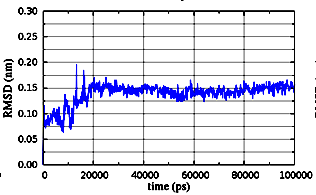 |
| 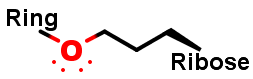  **44**  Log(D)=-1.55 | 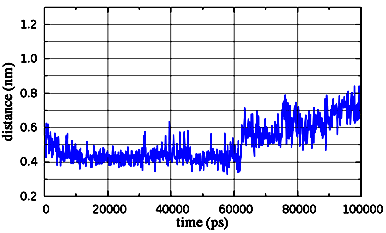 | 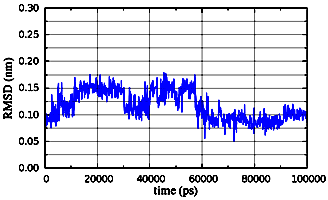 |

With these modifications the inhibitor cannot bind simultaneously in the active site and AdoMet site, and act as a transition state analogue (Fig 3).

| 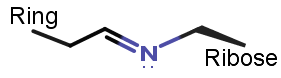  **45**  Log(D)=-0.42 | 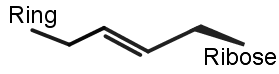  **46**  Log(D)=1.08 | 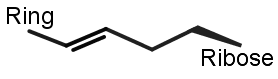  **47**  Log(D)=1.08 |
| --- | --- | --- |

**Table 3C. Changes that restrict mobility of the linker:**

| **Structure modification and its number.**  **LogD @pH=7.4** | **GROMACS MM/MD frames**  **Cys1226 ringC6 distance** | **GROMACS MM/MD frames**  **RMSD Ligand**  **relative to the first frame** |
| --- | --- | --- |
| 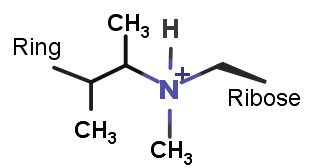  **48**  Log(D)=-2.84 | 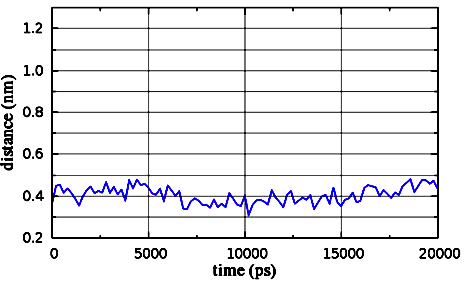 | 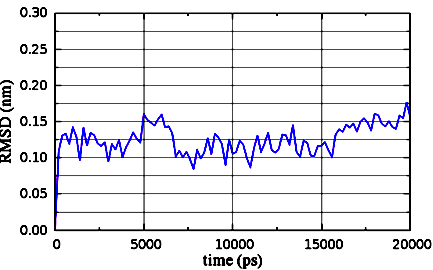 |
| 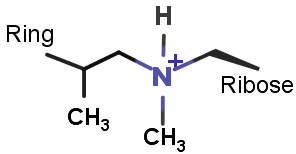  **49**  Log(D)=-2.99 | 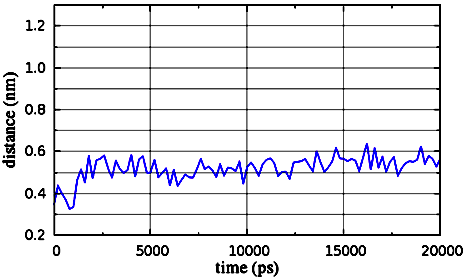 | 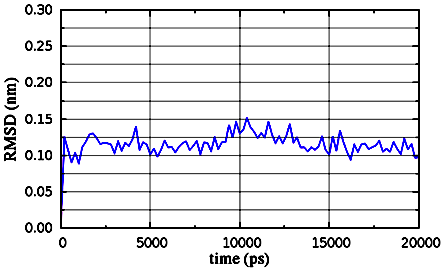 |
| 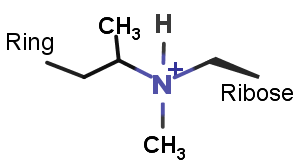  **50**  Log(D)=-2.92 | 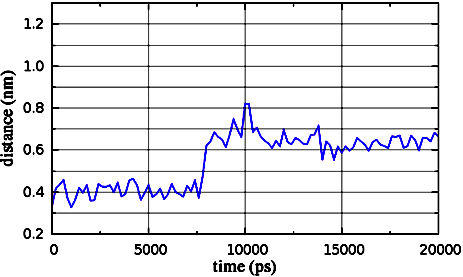 | 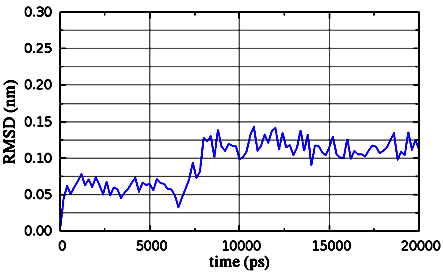 |
