# Supplementary material for: In silico design of the first DNA-independent mechanism-based inhibitor of mammalian DNA methyltransferase Dnmt1
Source: PLoS One. 2017 Apr 11;12(4):e0174410. doi: 10.1371/journal.pone.0174410 (PMC5388339; doi:10.1371/journal.pone.0174410)
Supplement: S4 Table — (DOC) [file pone.0174410.s004.doc]

In all tables, **the first column** shows the lead compound or its different modifications labeled with corresponding numbers. The LUMO values were calculated with program GAMESS using DFT B3LYP protocol as indicated in methods. **The second column** shows “Cys1226-carbon-6” distance plots. The plots can be used to evaluate likelihood for a mechanism-based suicide-inhibition. A distance below 4 Å indicates that active site Cys1226 and the target base are in a close Van der Waals contact that can support the formation of the covalent adduct and a mechanism-based suicide-inhibition (Fig 6). The **third column** shows RMSD values relative to the initial complex that was prepared using a rigid body docking protocol . Different binding orientations can be seen as large peaks or steps in plots of “average-ligand-RMSD” values. Distinct steps in RMSD plots represent distinct conformations of the ligand within the complex, while uniform RMSD plots with low variability represent a tightly bound inhibitor. All MM/MD simulations started with 20 nsec simulations. For more detailed description we used 100 nsec simulations. The modifications that give favorable LogD values and “Cys1226-carbon-6” distance plots are marked with red numbers.

**Table 4. Modifications in the target base ring**:

| **Structure modification and its number.** | **GROMACS MM/MD frames**  **Cys1226 ringC6 distance** | **GROMACS MM/MD frames**  **RMSD Ligand**  **relative to the first frame** |
| --- | --- | --- |
| 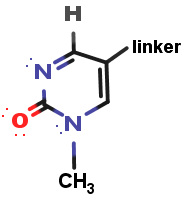  1-methyl-pirimidin-2-one  (lead compound  as reference)  LUMO=-1.4 eV | 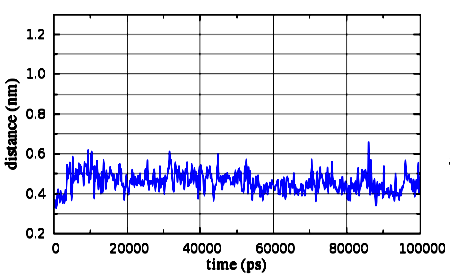 | 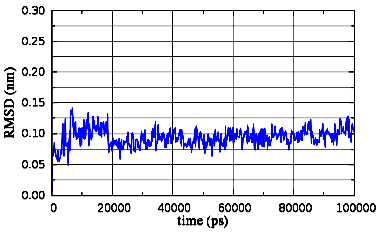 |
| 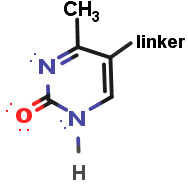  **51**  LUMO=-1.4 eV | With this modification the inhibitor cannot simultaneously bind to the active site and AdoMet site and act as a transition state analogue (Fig 3). | |
| 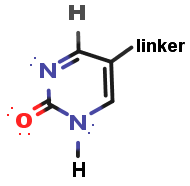  Pirimidine-2-one  **52**  LUMO=-1.4 eV | 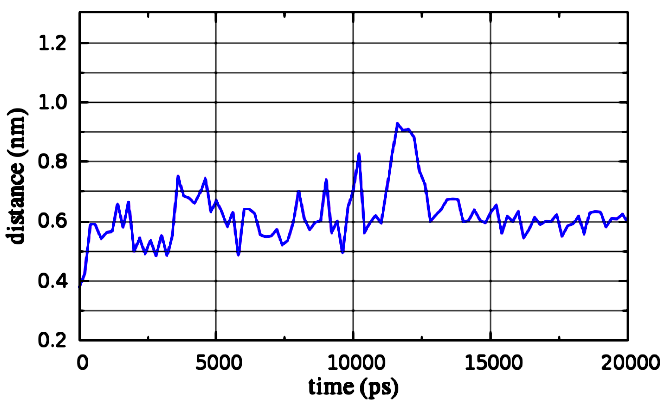 | 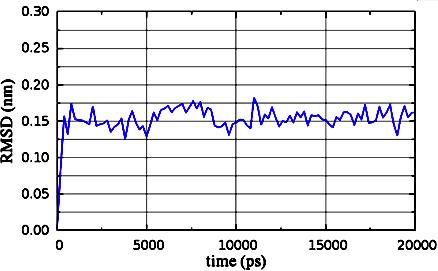 |
| 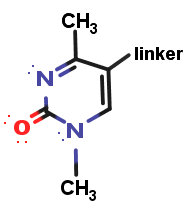  **53**  LUMO=-1.5 eV | 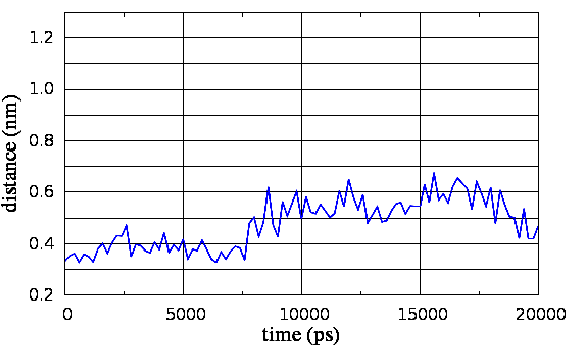 | 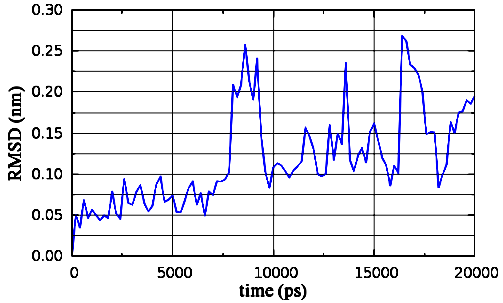 |
| 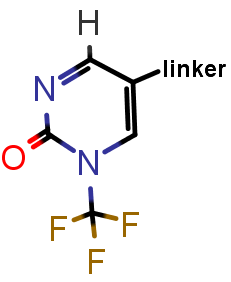  **54**  LUMO=-2.3 eV | 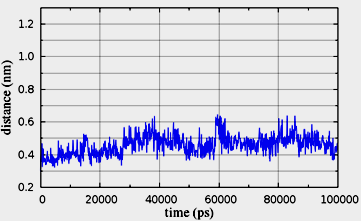 | 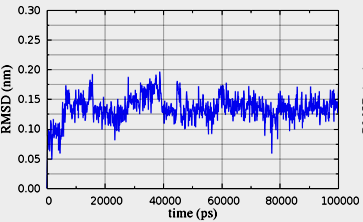 |
| 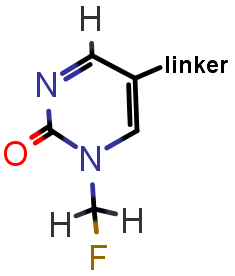  **55**  LUMO=-2.1 eV | 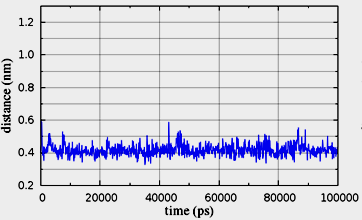 | 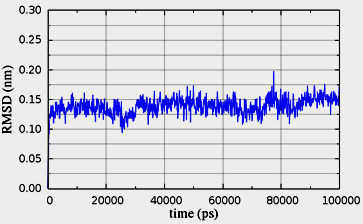 |
| 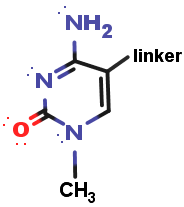  1-methyl-cytosine  **56**  LUMO=-0.9 eV | 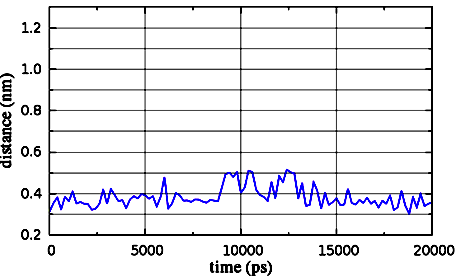 | 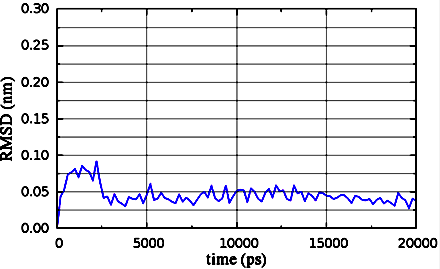 |
| 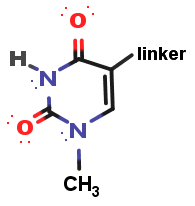  1-methyl-uracil  **57**  LUMO=-0.6 eV | 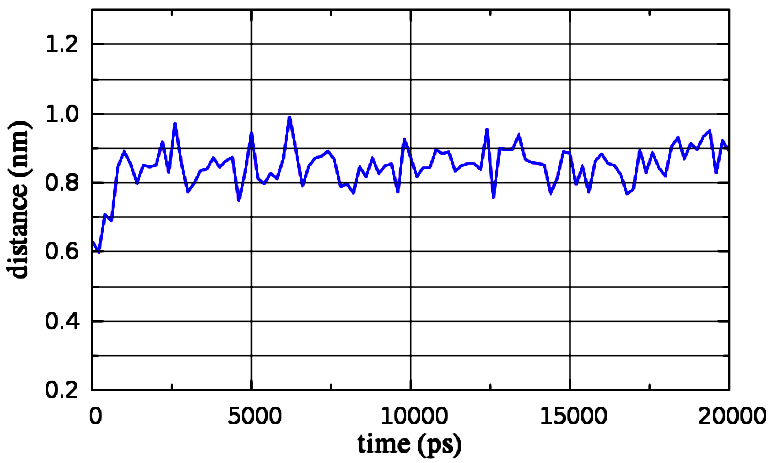 | 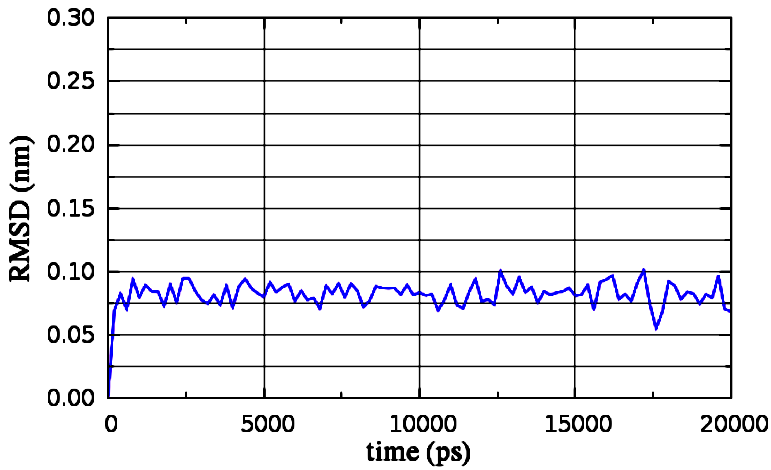 |
